# Truncating mutation in the autophagy gene *UVRAG* confers oncogenic properties and chemosensitivity in colorectal cancers

**DOI:** 10.1038/ncomms8839

**Published:** 2015-08-03

**Authors:** Shanshan He, Zhen Zhao, Yongfei Yang, Douglas O'Connell, Xiaowei Zhang, Soohwan Oh, Binyun Ma, Joo-Hyung Lee, Tian Zhang, Bino Varghese, Janae Yip, Sara Dolatshahi Pirooz, Ming Li, Yong Zhang, Guo-Min Li, Sue Ellen Martin, Keigo Machida, Chengyu Liang

**Affiliations:** 1 Department of Molecular Microbiology and Immunology, Keck Medical School, University of Southern California, Los Angeles, California 90033, USA; 2 Department of Radiology, Keck Medical School, University of Southern California, Los Angeles, California 90033, USA; 3 Key Laboratory of Carcinogenesis and Translational Research, Department of Colorectal Surgery, Peking University Cancer Hospital & Institute, Beijing 100142, China; 4 Department of Surgical Oncology, the First Affiliated Hospital of Medical College, Xi’an Jiaotong University, Xi’an 710061, China; 5 Graduate Center for Toxicology, Markey Cancer Center, University of Kentucky College of Medicine, Lexington, KY 40506, USA; 6 Department of Pathology, Keck Medical School, University of Southern California, Los Angeles, California 90033, USA

## Abstract

Autophagy-related factors are implicated in metabolic adaptation and cancer metastasis. However, the role of autophagy factors in cancer progression and their effect in treatment response remain largely elusive. Recent studies have shown that UVRAG, a key autophagic tumour suppressor, is mutated in common human cancers. Here we demonstrate that the cancer-related UVRAG frameshift (FS), which does not result in a null mutation, is expressed as a truncated UVRAG^FS^ in colorectal cancer (CRC) with microsatellite instability (MSI), and promotes tumorigenesis. UVRAG^FS^ abrogates the normal functions of UVRAG, including autophagy, in a dominant-negative manner. Furthermore, expression of UVRAG^FS^ can trigger CRC metastatic spread through Rac1 activation and epithelial-to-mesenchymal transition, independently of autophagy. Interestingly, UVRAG^FS^ expression renders cells more sensitive to standard chemotherapy regimen due to a DNA repair defect. These results identify *UVRAG* as a new MSI target gene and provide a mechanism for UVRAG participation in CRC pathogenesis and treatment response.

## Abstract (web summary)

Some colon carcinomas with microsatellite instability carry a frameshift mutation in a tumour suppressor UVRAG. Here the authors show that mutant UVRAG triggers colorectal cancer by antagonizing the activity of normal UVRAG in autophagy and chromosomal stability, but also sensitizes the cancer to DNA damage-inducing chemotherapeutic drugs.

## Graphical abstract

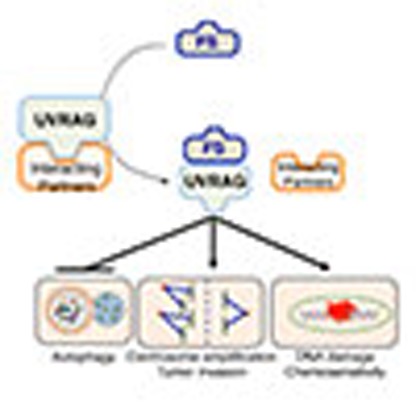

Colorectal cancer (CRC) remains one of the most widespread malignancies worldwide^1^. Approximately 15% of sporadic CRC and 90% of Lynch syndrome (hereditary nonpolyposis colorectal cancer) exhibit a microsatellite instability (MSI) phenotype, caused by a deficiency in DNA mismatch repair (MMR) that progresses with a high rate of insertions/deletions to repetitive DNA sequences, termed microsatellites^2^. Increasing evidence suggests that MMR deficiency *per se* is not sufficient to drive cell transformation and tumorigenesis, but that microsatellite mutations in a limited number of target genes might be positively selected during tumour development and underlie MSI-associated pathogenesis and treatment response^3^,^4^. Frameshift (FS) mutations of several autophagy-related genes, including Atg2b, Atg5, Atg9b, Atg12 and UVRAG (ultraviolet irradiation resistance-associated gene)^5^,^6^,^7^, were recently reported in gastric cancer and CRC with MSI. Nevertheless, the functional consequences and key molecular events downstream of these mutations have not been extensively investigated.

Our previous studies have established UVRAG as a critical regulator of intracellular membrane trafficking, including autophagy and chromosomal stability^6^,^8^,^9^,^10^,^11^,^12^,^13^,^14^,^15^,^16^. UVRAG contains four functional domains, that is, a proline-rich domain, a lipid-binding C2 domain, a Beclin1-binding coiled-coil domain (CCD) and a C-terminal domain presumed to be unstructured and involved in centrosome integrity and DNA damage repair (Supplementary Fig. 1a)^12^,^17^. Importantly, all the different activities of UVRAG are functionally independent, suggesting biological interaction and coordinated regulation of the different processes under diverse environmental cues. Although most cellular studies to date have considered *UVRAG* as a tumour suppressor in human cancers^18^, the genetic linkage of *UVRAG* mutations in major tumour types and the significance of these mutations in tumour pathogenesis remains less understood.

Here we show that MSI CRCs with the FS mutation in *UVRAG* express a truncated UVRAG protein, referred to here as UVRAG^FS^. In addition to losing the wild-type (WT) UVRAG functions, this nonsense mutant acts as a dominant-negative mutant and contributes to the oncogenesis and tumour metastasis of CRC, likely by antagonizing the activity of UVRAG^WT^ as a tumour suppressor. UVRAG^FS^ expression also increases the sensitivity to anticancer agents such as 5-fluorouracil (5-FU), oxaliplatin and irinotecan, routinely prescribed as adjuvant therapies for CRC patients. Our data thus identified the underlying pathogenic mechanisms beyond autophagy that are associated with UVRAG^FS^-positive cancers and suggest that expression of UVRAG^FS^ might also be a predictive factor for chemotherapy response.

## Results

### UVRAG A_10_ DNA microsatellite mutation in MSI CRC

**Figure 1:**
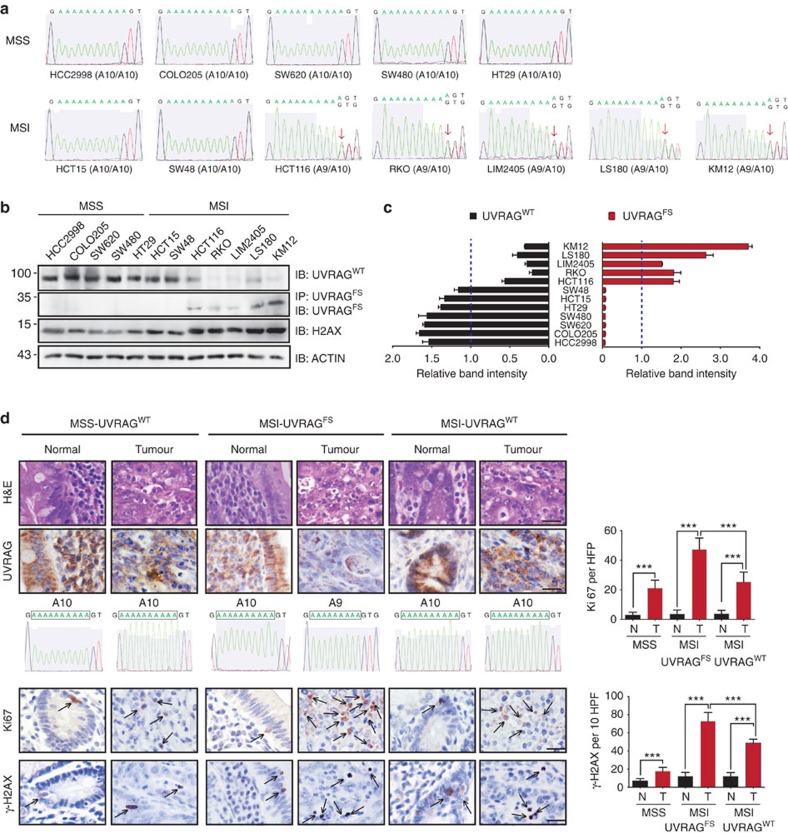
Identification of *UVRAG* FS mutation in CRC cell lines and primary tumours. (**a**) Sequencing analysis of *UVRAG* at the location of the A_10_ repeat in MSS (HCC2998, COLO205, SW620, SW480 and HT29) and MSI (HCT15, SW48, HCT116, RKO, LIM2405, LS180 and KM12) CRC cell lines. Arrows indicate the heterozygous deletion of one A in *UVRAG* A_10_ in MSI cell lines. (**b**,**c**) Wild-type (WT) and FS mutant UVRAG protein expression in MSS and MSI CRC cell lines. Whole-cell lysates (WCL) of MSS and MSI CRC cell lines were immunoprecipitated with anti-UVRAG^FS^ followed by immunoblotting with anti-UVRAG^FS^, or they were directly probed with antibodies targeting UVRAG^WT^ or γ-H2AX. Actin served as a loading control. Densitometric quantification of protein expression is shown in (**c**). Dash lines indicate average band intensities of all the tested cell lines. Note reduced UVRAG^WT^ expression in MSI CRC cells expressing UVRAG^FS^. (**d**) H&E (first row) and immunohistochemical analysis of UVRAG (second row), Ki67 (fourth row), and γ-H2AX (5th row) in paired human primary CRC specimen obtained from three separate patients with their corresponding status of *UVRAG* FS mutation (third row) provided. The bar plots (right) are the quantification of the levels of Ki67 and γ-H2AX (denoted by arrows) in the paired tissues with WT or mutant UVRAG. HPF, high-power field. ***P<0.001 (Mann–Whitney test); Scale bar ,50 μm.

The human *UVRAG* gene contains a tract of A_10_ mononucleotide repeats in exon 8, spanning codons 234–237 (5′-AAA AAA AAA AGT-3′; Supplementary Fig. 1a,b). Using seven MSI^+^ CRC cell lines (HCT15, HCT116, KM12, LIM2405, LS180, RKO and SW48) and genomic sequencing, we confirmed, as reported previously^6^,^7^,^16^, the heterozygous FS deletion of one nucleotide (A) in the *UVRAG* A_10_-coding repeat in most tested MSI^+^ CRC cells, with the exception of HCT15 and SW48. In contrast, MSS (microsatellite stable) cells, including COLO205, HCC2998, HT29, SW480 and SW620, contained only WT coding repeats (Fig. 1a). The FS mutation was predicted to produce a premature stop codon and therefore a truncated UVRAG^7^ (referred here as UVRAG^FS^; Supplementary Fig. 1a,b). To assess whether this mutation is indeed expressed in MSI cells, we generated an antibody specifically recognizing UVRAG^FS^, but not UVRAG^WT^, using the FS-derived neopeptide (_234_KKKVNACS_241_) as antigen (Supplementary Fig. 1b,c). UVRAG^FS^ expression was detected in all MSI cell lines carrying the FS mutation, but not in MSI or MSS cells that are WT for *UVRAG* (Fig. 1b). Notably, the overall expression of UVRAG^WT^ was diminished in MSI cells with the FS mutation (Fig. 1b), and the levels of UVRAG^FS^ were inversely correlated with the expression of UVRAG^WT^ in all tested cell lines (Fig. 1c). This was consistent with the UVRAG expression profile from the CRC cell lines of the NCI-60 panel^19^. Therein, a significant reduction of UVRAG^WT^ expression was detected in UVRAG^FS^-positive KM12 and HCT116 CRC cells compared with other CRC cells without UVRAG^FS^ (Supplementary Fig. 1d). In addition, the *UVRAG* FS mutation was present in one of the four analysed cases of human primary CRC with MSI (fourth column in Fig. 1d), but not in primary MSS CRC or in normal colorectal mucosa (Fig. 1d, Supplementary Table 1). This is in line with a previous report^2α^ that evaluated the mutation frequencies in 137 genes in MSI cancers, revealing the high frequency of the A_10_ *UVRAG* FS mutation that was found in 33% CRC, 8% endometrial and 7.8% gastric cancers with MSI (Supplementary Fig. 1e). Whole-genome sequencing analysis of a large cohort of gastric cancers (Pfizer and UHK; *n*=100) also confirmed the presence of the *UVRAG* FS mutation in MSI gastric cancer (40%)^20^. Collectively, these results indicate that the frameshift *UVRAG* mutation is likely selected and is expressed as a truncated UVRAG protein in MSI tumours.

### Oncogenic property of the *UVRAG* ^FS^ mutation

**Figure 2:**
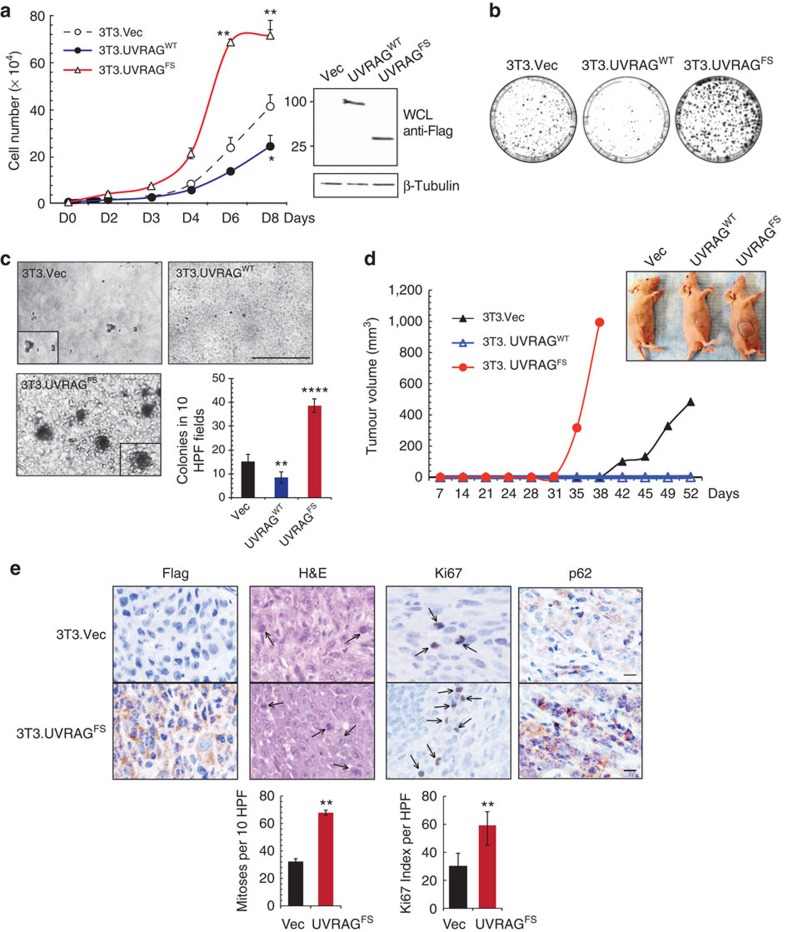
Cell transformation and oncogenic effect of UVRAG^FS^ mutant. (**a**) NIH3T3 cells stably expressing empty vector, UVRAG^WT^, and UVRAG^FS^ (10^4^) were seeded and counted over time in triplicate. Values are the means±s.d. (*n*=4). Flag-tagged UVRAG expression is shown by western blot and β-tubulin serves as a loading control. *P<0.05; **P<0.01. (**b**) NIH3T3 cells described above were plated at low density (2,500 cells per 10-cm plate), grown for 14 days then fixed and stained with crystal violet. (**c**) Anchorage-independent growth induced by UVRAG^FS^. NIH3T3 cells in (**a**) were seeded in 0.3% top agar and incubated for 20 days. UVRAG^FS^-expressing cells formed larger and greater number of colonies in soft agar. Representative images of colonies are shown and the quantitative results of colony numbers were obtained from 10 randomly chosen HPF. Data represent the means±s.d. (*n*=4). **P<0.01; ****P<0.0001. Scale bar, 500 μm. (**d**) UVRAG^FS^-associated oncogenesis in nude mouse model. UVRAG^FS^-NIH3T3 cells from (**a**) were subcutaneously injected into flanks of nude mice, and tumour growth was measured over time. Circles indicate xenograft tumours at day 38 after inoculation. Data shown are representative of three separate experiments. (**e**) Immunohistochemistry staining of 3T3-tumours with the indicated antibodies and their quantification in bar graphs (bottom). Arrows denote the mitotic and Ki67^+^ proliferating cells in the tumour. **P<0.01. Scale bar, 50 μm.

To probe whether the UVRAG^FS^ mutant represents a mere loss of WT function^11^ as occurs with most other tumour suppressors, or imparts oncogenic properties, we established MSS SW480 and MSI HCT116 cell lines stably expressing Flag-tagged UVRAG^WT^ and UVRAG^FS^ at equivalent levels (Supplementary Fig. 2a,d). UVRAG^FS^-transduced cells showed increased proliferation and enhanced anchorage-independent growth in soft agar (Supplementary Fig. 2a–e), independently of the tissue of origin (Supplementary Fig. 2f,g). Subcutaneous transplantation in athymic nude mice of UVRAG^FS^ SW480 cells resulted in tumour formation with accelerated kinetics (Supplementary Fig. 2c). To further test whether expression of UVRAG^FS^ is sufficient to transform noncancerous cells, we used NIH3T3 mouse embryonic fibroblasts stably expressing UVRAG^WT^ or UVRAG^FS^ (Fig. 2a). Compared with control (3T3.Vec), UVRAG^FS^–3T3 cells had elevated growth rate, formed larger colonies when plated at low density and induced anchorage-independent growth, whereas UVRAG^WT^ exerted the opposite effects (Fig. 2a–c). The tumour growth rate and mean tumour volume were drastically increased when 3T3–UVRAG^FS^ cells were injected into nude mice (Fig. 2d). Immunohistological analyses of tumour xenografts showed UVRAG^FS^ expression and enhanced mitotic index and number of Ki67^+^ (proliferating) cells in UVRAG^FS^ tumours (Fig. 2e). CRC primary tumours with the FS mutation also had increased Ki67 staining (Fig. 1d). Altogether, these data indicate a strong association of the cancer-derived UVRAG^FS^ with a tumorigenic phenotype.

### Dominant-negative effect of UVRAG^FS^ on autophagy activation

**Figure 3:**
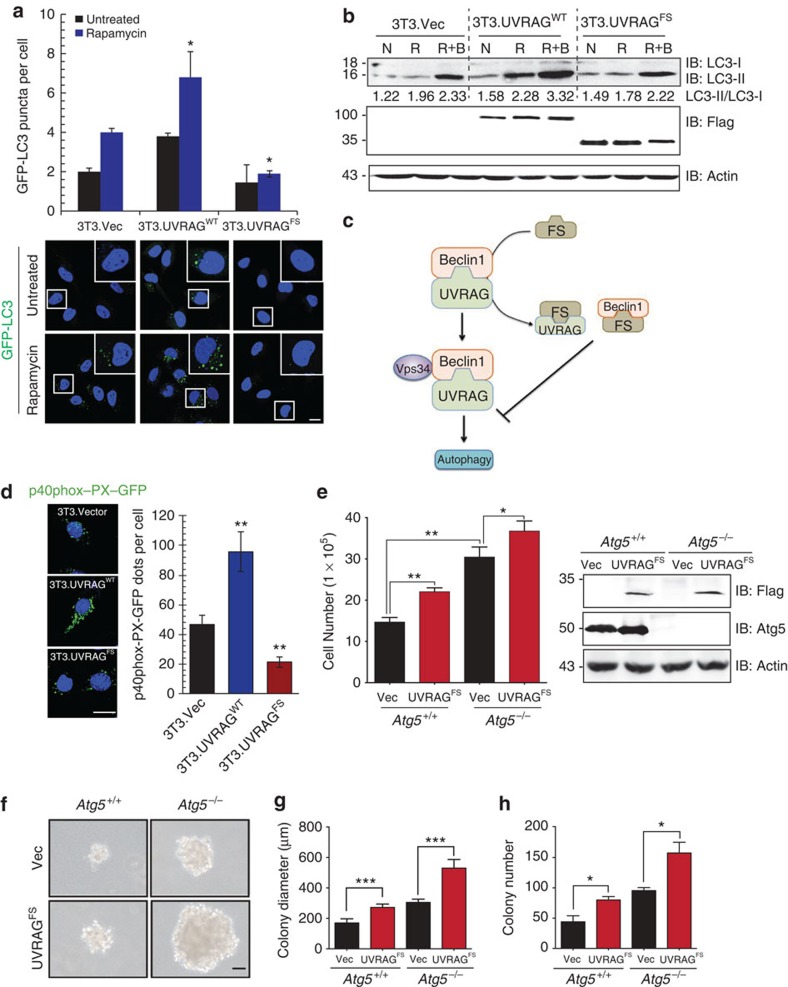
UVRAG^FS^ inhibits cellular autophagy in a dominant-negative manner. (**a**) NIH3T3 cells stably expressing vector, UVRAG^WT^, and UVRAG^FS^ were transfected with GFP–LC3 and treated with rapamycin (100 nM). GFP–LC3 puncta per cell were quantified as shown in representative images shown. Data represent the means±s.d. (*n*=6). *P<0.05. Scale bar, 10 μm. (**b**) Western blot analysis and densitometric quantification (underneath the blot) of the LC3-II/LC3-I ratios in NIH3T3 cells treated with rapamycin in the presence or absence of Bafilomycin A_1_ (100 nM). N, normal condition; R, rapamycin; R+B, rapamycin+Bafilomycin A_1_. (**c**) Schematic depiction of the dominant-negative action of UVRAGFS on the UVRAG-Beclin1 interaction by sequestering both. (**d**) UVRAG^FS^ inhibits Beclin1-associated VPS34 kinase activity. NIH3T3 cells from (**a**) were transfected with p40(phox)-PX-GFP (to monitor phosphatidylinositol 3-phosphate formation). At 16 h post-transfection, cells were subjected to confocal microscopy and p40(phox)-PX-GFP puncta per cell were quantified. Data represent the means±s.d. (*n*=3). **P<0.01. Scale bar, 10 μm. (**e**) UVRAG^FS^ promotes cell proliferation in *Atg5*-knockout iMEFs. *Atg5*^+/+^ and *Atg5*^−/−^ iMEF cells stably expressing vector and UVRAG^FS^ were seeded and counted in triplicate on day 8. Values are mean±s.d. (*n*=3). UVRAG and Atg5 expression was assessed by western blot with actin serving as a loading control. *P<0.05; **P<0.01. (**f**–**h**) Anchorage-independent growth of *Atg5*-knockout iMEFs expressing UVRAG^FS^. Note the larger and greater number of colonies in UVRAG^FS^-expressing cells. Colony diameters (**g**) and numbers (**h**) were quantified from 20 random HPFs. Data are the means±s.d. (*n*=3). *P<0.05; ***P<0.001. Scale bar, 50 μm.

UVRAG^FS^ retains the N-terminal proline-rich and C2 domains, and the partial CCD required for Beclin1-mediated autophagy (Supplementary Fig. 1a)^10^,^12^,^21^,^22^,^23^. To determine whether UVRAG^FS^ retained its autophagy activity, we measured the subcellular distribution of the autophagy marker green-fluorescent protein (GFP)-LC3 and the levels of the autophagosome-associated lipidated LC3 (LC3-II)^24^,^25^ in noncancerous NIH3T3 cells. As shown previously^10^,^12^,^26^, UVRAG^WT^ or rapamycin markedly promoted autophagy, as evidenced by increased GFP–LC3 puncta per cell, increased LC3-II conversion and increased response to the late-stage autophagy inhibitor Bafilomycin A_1_ (Fig. 3a,b). In sharp contrast, UVRAG^FS^ did not demonstrate any proautophagic activity. Furthermore, UVRAG^WT^ autophagy-promoting activity was abrogated when UVRAG^FS^ was added to the cells dose dependently (Supplementary Fig. 3a). UVRAG interacts with Beclin1 through their respective CCD, resulting in activation of Beclin1-associated Vps34 kinase^27^. On UVRAG^FS^ expression, the endogenous association between UVRAG^WT^ and Beclin1 was diminished, and UVRAG^FS^ was able to sequester the Beclin1 and UVRAG proteins *in vivo*, in line with its dominant-negative effect (Supplementary Fig. 3b, Fig. 3c). Accordingly, Vps34 enzymatic activity was significantly reduced in UVRAG^FS^ cells (Fig. 3d), as illustrated by decreased punctate staining of the Vps34 kinase product, phosphatidylinositol 3-phosphate^28^. Impaired autophagy was also observed *in vivo* in NIH3T3 tumour xenografts expressing UVRAG^FS^ (Fig. 2e), showing increased levels of p62, an autophagic substrate^29^. To explore whether autophagy inhibition underlies UVRAG^FS^-mediated oncogenesis, we examined the transforming effect of UVRAG^FS^ in autophagy-null Atg5-deficient MEFs^29^. UVRAG^FS^ promoted cell proliferation (Fig. 3e) and colony growth in soft agar (Fig. 3f–h), irrespective of the autophagy status. These data support a direct role of UVRAG^FS^ in promoting tumorigenesis independently of autophagy.

### UVRAG^FS^ induces chromosomal instability and centrosome amplification

**Figure 4:**
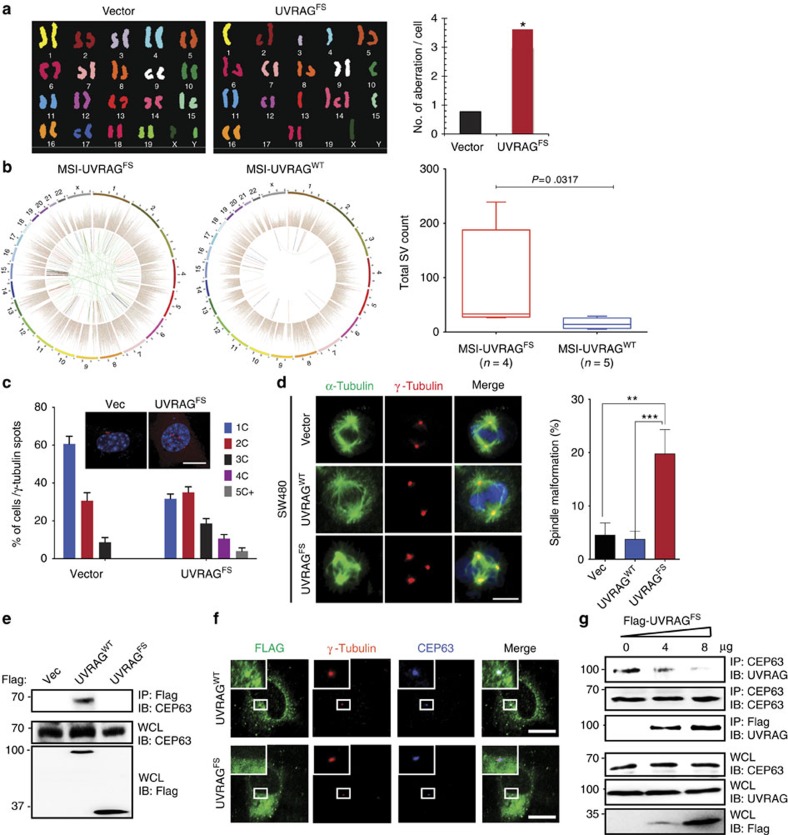
UVRAG^FS^ promotes chromosomal instability and centrosome amplification. (**a**) Representative SKY analysis of mouse embryonic stem (ES) cells expressing vector or Flag-UVRAG^FS^. Average chromosomal aberrations per cell were quantified. *P<0.05, Wilcoxon Signed-rank Test. (**b**) Chromosomal rearrangement and single-nucleotide variants (SNVs) in UVRAG^FS^ gastric cancer. Representative Circos plots of the UVRAG^WT^ and UVRAG^FS^ subtypes of MSI gastric cancer in the Pfizer and UHK cohorts^20^. The inner circle denotes chromosomal structural variants: ITX (intrachromosomal translocation), blue; DEL (deletion), red; CTX (interchromosomal translocation), green; INV (inversions), pink; Tandem duplication, black. In the second circle, each dot denotes one somatic SNV, coloured according to six mutation types: T>G, pink; T>C, green; T>A, gray; C>G, black; C>A, blue; C>T, red. The outer circle denotes 23 chromosomes. Boxplot represents the total chromosomal structure variants (SV) count in the UVRAG^WT^ and UVRAG^FS^ MSI gastric cancers (Mann–Whitney test). (**c**) UVRAG^FS^ induces centrosome amplification. SW480.Vector and SW480.UVRAG^FS^ cells with different centrosome numbers were immunostained for γ-Tubulin and DAPI and quantified (Data are the means±s.d., *n*=200 cells obtained by gathering data from three independent experiments). (**c**) centrosome. Scale bar, 10 μm. (**d**) Representative confocal images of spindle malformation in mitotic Vector, UVRAG^WT^, and UVRAG^FS^ SW480 cells co-stained with anti-γ-Tubulin (red) and anti-α-Tubulin (green) for the mitotic asters. The percentages of cells with disorganized spindle were quantified. Data are the means±s.d. (*n*=200 cells obtained by pooling data from three independent experiments). Scale bar, 10 μm. (**e**) UVRAG^FS^ is defective in CEP63 binding. Whole-cell lysates (WCL) of 293T transfected with Flag-UVRAG^WT^ or Flag-UVRAG^FS^ were immunoprecipitated with anti-Flag followed by IB with anti-CEP63. (**f**) Representative image showing dissociation of UVRAG^FS^ from the centrosome. HeLa cells expressing Flag-UVRAG^WT^ or Flag-UVRAG^FS^ were stained with anti-Flag (green), anti-γ-Tubulin (red), and anti-CEP63 (blue). Scale bar, 10 μm. (**g**) UVRAG^FS^ inhibits UVRAG-CEP63 interaction. The 293T cells were transfected with increasing amounts of Flag-UVRAG^FS^. WCL were immunoprecipitated with anti-CEP63 or anti-Flag, followed by immunoblotting with anti-UVRAG or anti-CEP63 as indicated.

Because the role of UVRAG in cancer has been linked to its ability to maintain chromosomal stability^17^, we investigated the effect of UVRAG^FS^ on overall chromosomal stability in genetically stable mouse embryonic stem cells. Spectral karyotyping analysis showed that, unlike control cells that were mostly diploid, *UVRAG*^*FS*^-embryonic stem cells were highly heterogeneous with respect to both structural and numerical aberrations as compared with the vector control (Fig. 4a, Supplementary Fig. 4a) with a greater than sevenfold increase in aneuploidy in UVRAG^FS^ cells (Supplementary Fig. 4b). These results indicate that UVRAG^FS^ elicits severe chromosomal instability and aneuploidy. To validate this, we analysed the Pfizer and UHK cohort^20^ of gastric cancers, and observed significantly enhanced chromosomal rearrangement in UVRAG^FS^ MSI gastric cancers as compared with UVRAG^WT^ MSI gastric cancers (Fig. 4b). In fact, UVRAG^FS^ gastric cancers had substantially more protein-altering mutations and single-nucleotide variants than UVRAG^WT^ MSI and MSS gastric cancers (Supplementary Fig. 4c). Moreover, the FS mutation appeared to be more frequent in gastric cases with advanced tumour, node, metastasis stage (Supplementary Fig. 4d). Thus, UVRAG^FS^ may predispose MSI cancers to increased genetic instability and cancer progression.

UVRAG^WT^ has been shown to associate with the centrosome protein CEP63 (ref. 17), contributing to chromosomal stability by preventing centrosome overduplication^17^. UVRAG^FS^ expression in SW480 cells was sufficient to induce a marked increase in the incidence and degree of centrosome amplification compared with control (Fig. 4c). Consistent with the consensus that centrosome amplification causes erroneous chromosomal segregation^30^, we detected spindle malformation, chromosomal missegregation and prolonged mitosis in UVRAG^FS^ clones, whereas UVRAG^WT^ clones behaved in the opposite manner (Fig. 4d, Supplementary Fig. 5a). Unlike WT, UVRAG^FS^ was unable to associate with CEP63 (Fig. 4e), failing to colocalize with CEP63 and the centrosome marker, γ-Tubulin (Fig. 4f). UVRAG^FS^ disrupted UVRAG^WT^-CEP63 interaction (Fig. 4g) and displaced UVRAG from the centrosome in a dominant-negative manner (Supplementary Fig. 5b). These results indicate that centrosome amplification induced by UVRAG^FS^ may play a role in UVRAG^FS^-associated chromosomal aneuploidies.

### UVRAG^FS^ promotes cell invasion and metastasis outgrowth

**Figure 5:**
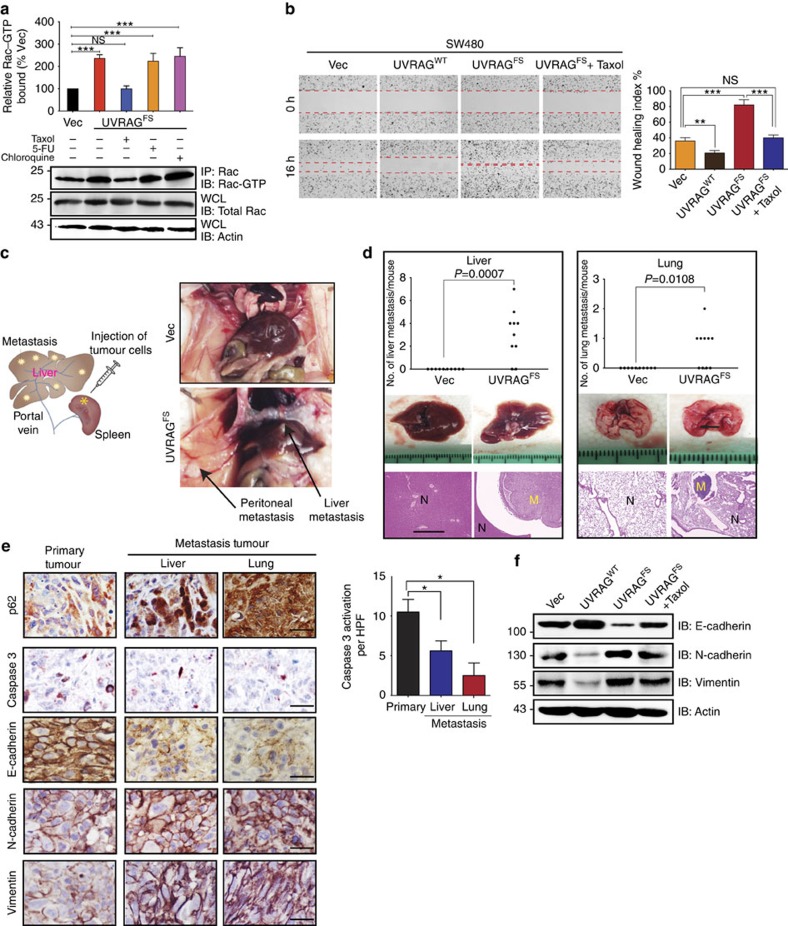
UVRAG^FS^ activates Rac-1 and promotes tumour metastasis *in vitro* and *vivo*. (**a**) Rac-1 activation by UVRAG^FS^. Western blot shows a pull-down experiment to detect GTP-bound Rac1 in SW480.UVRAG^FS^ cells and on drug treatment. Histogram shows quantification from three independent experiments. (**b**) Representative images of scratch-wound healing exhibit the motility of SW480.UVRAG^FS^ cells. Cell motility into the wound area was taken at 0 and 16 h as marked by red lines. Wound-healing index was quantified (right). Data are the means±s.d. (*n*=3). **P<0.01; ***P<0.001; NS, not significant. Histogram shows quantification from three independent experiments. (**c**,**d**) UVRAG^FS^ enhances tumour metastasis in mice inoculated by intrasplenic injection of SW480.Vector and SW480.UVRAG^FS^ cells. Schematic depiction of the procedure (**c**, left) and representative images (**c**, right) of upper abdominal organs at 8-week post-injection are shown (**c**). The number of metastatic nodules (liver and lung) was quantified (**d**). H&E staining was performed on serial sections of metastatic tumours (M) and normal (N) liver and lung are shown below. Scale bar, 1 mm. Arrows in **c** represent the metastasis foci. Results are representative of 10 mice per group. Fisher’s exact test was used. (**e**) Immunohistochemistry analysis of autophagy, apoptosis and EMT status of primary tumours (left panel) and metastasis nodules (right panel). The bar graphs represent the quantification of the indicated protein markers. Data are the means±s.d. (*n*=3). *P<0.05. Scale bar, 50 μm. (**f**) Western blot of the EMT-related protein expression in SW480 cells expressing UVRAG^FS^ and on Taxol treatment.

Centrosome amplification *per se* has been shown to promote cell invasion through inappropriate microtubule nucleation and Rac-1 activation^31^, a small GTPase important for the control of cell invasiveness and metastasis^32^,^33^. Indeed, pull-down assay in UVRAG^FS^ SW480 cells detected a more than twofold Rac1 activation, which could be blocked by Taxol, but not by the autophagy inhibitor chloroquine or the anticancer reagent 5-FU (Fig. 5a), indicating a requirement for dynamic microtubules. Consistent with increased Rac1 activation, UVRAG^FS^ enhanced the cell motility of SW480 cells in a wound-healing assay, which was inhibited by Taxol (Fig. 5b). It also enhanced HCT116 cell migration through a collagen matrix, whereas UVRAG^WT^ exerted an inhibitory effect (Supplementary Fig. 6a,b). Spleen injection of non-metastatic SW480 cells expressing UVRAG^FS^ into nude mice resulted in a higher incidence of liver metastasis and a greater number of colonization in the lungs, kidney and peritoneum, whereas no colonization was found in the control group (Fig. 5c,d, Supplementary Fig. 6c). UVRAG^FS^-induced tumour metastases were confirmed in an independent mouse metastasis model with SW480 cells expressing GFP–UVRAG^FS^, as determined by bioluminescence imaging of metastatic lesions (Supplementary Fig. 6d). These results indicate that UVRAG^FS^ enhances the metastatic capacity of CRC cells.

Autophagy has been postulated to be exploited by metastatic tumours to survive unfavourable conditions^34^. Nevertheless, UVRAG^FS^-metastatic tumours displayed higher levels of p62 than primary tumours, indicative of suppressed autophagy (Fig. 5e). Moreover, UVRAG^FS^ metastatic tumours exhibited decreased apoptosis, as shown by decreased caspase 3 activation (Fig. 5e). Hence, in this context, autophagy is not the driving mechanism for metastatic colonization in CRC. Nevertheless, we observed other pathological differences that may account for increased metastasis on UVRAG^FS^ expression. The colonized CRC tumours had reduced levels of the epithelial cell marker E-cadherin but increased levels of the mesenchymal markers, N-cadherin and vimentin (Fig. 5e), suggesting an induction of epithelial-mesenchymal transition (EMT) in the process of colonization. Indeed, expression of UVRAG^FS^ in SW480 cells downregulated E-cadherin and upregulated N-cadherin and vimentin, whereas expression of UVRAG^WT^ had the opposite effect (Fig. 5f). Importantly, UVRAG^FS^-associated EMT was efficiently reverted by Taxol without affecting Taxol-induced cell death (Fig. 5f, Supplementary Fig. 6f). Consistent with our *in vitro* observations, the primary MSI colon tumour with UVRAG^FS^ exhibited elevated expression of N-cadherin and vimentin, along with significant reduction in E-cadherin levels, which were not detected in tumours with UVRAG^WT^ (Supplementary Fig. 6e). These results indicate that UVRAG^FS^ expression, which triggers centrosome amplification and Rac1 activation, can activate the EMT program and promote cell invasion and tumour metastasis.

### UVRAG^FS^ affects CRC response to chemotherapy

**Figure 6:**
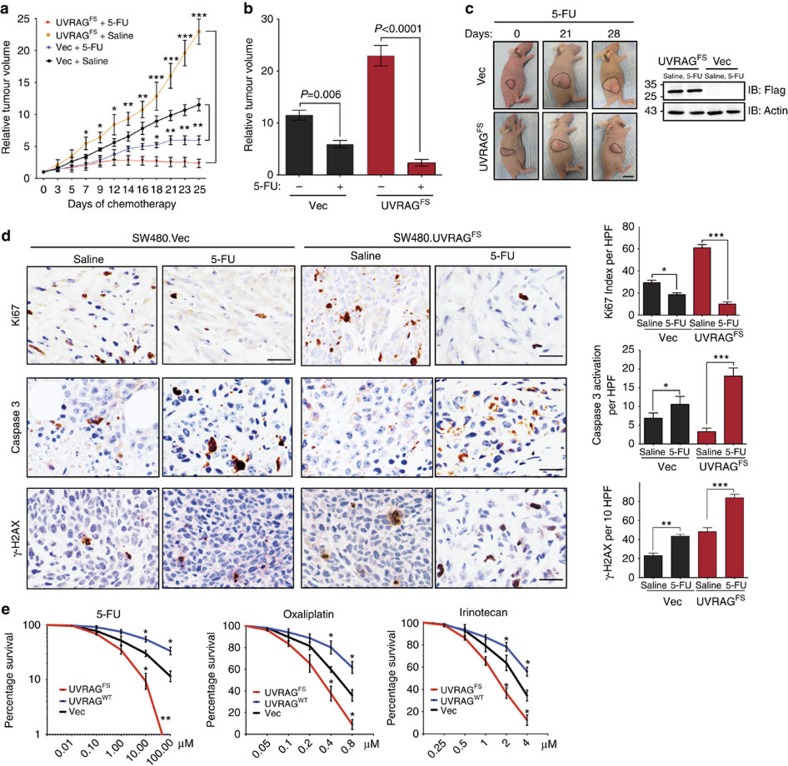
UVRAG^FS^ sensitizes CRC to DNA damage-inducing chemotherapy. (**a**) Mice bearing SW480.Vec and SW480.UVRAG^FS^ tumours were treated with saline or 5-FU over day 1–30 when the tumour volume reached 200 mm^3.^ Mean relative tumour volume (*n*=10), is expressed compared with tumour volumes on day 1. *P<0.05; **P<0.01; ***P<0.001. (**b**) Relative tumour volume for individual mice treated in **a** at day 25. (**c**) Representative images of mice bearing SW480-xenografts on the day 0, 21–28 of chemotherapy (left). Western blots showed UVRAG^FS^ expression in representative tumours. Scale bar, 5 mm. (**d**) Immunohistochemical analysis of SW480-xenografts harvested from mice treated for 28 continuous days with 5-FU. Representative sections were stained (left) as indicated and staining-positive cells were quantified (right) as means±s.d. Scale bar, 10 μm. *P<0.05; **P<0.01; ***P<0.001. (**e**) SW480.Vector, SW480.UVRAG^WT^ and SW480.UVRAG^FS^ were treated with the indicated doses of 5-FU, Oxaliplatin and Irinotecan, followed by colony survival assay. Data are the means±s.d. (*n*=3). *P<0.05; **P<0.01.

**Figure 7:**
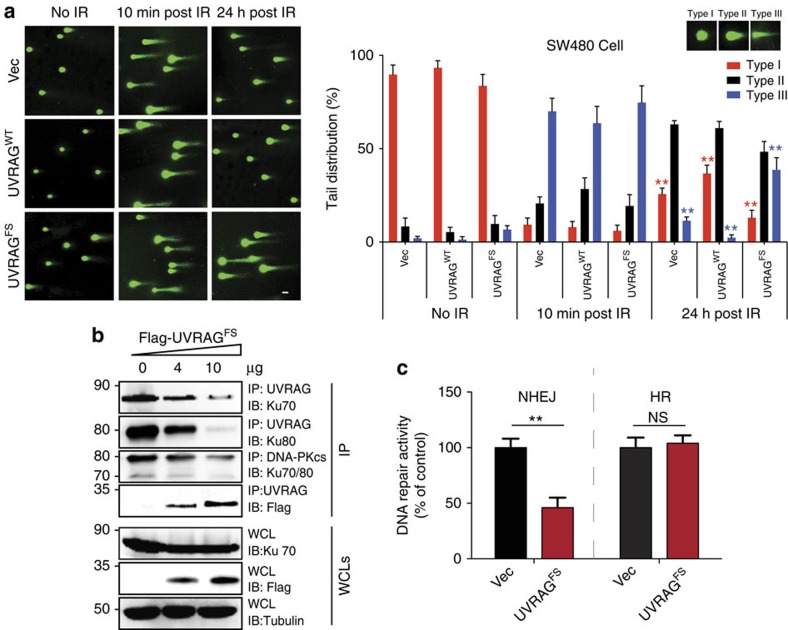
UVRAG^FS^ inhibits NHEJ repair. (**a**) Neutral comet assay shows a delay of DNA DSBs repair in UVRAG^FS^ cells. SW480 cells stably expressing empty vector (first row), UVRAG^WT^ (second row) or UVRAG^FS^ (third row) were treated with 1 Gy IR. The DNA damage levels of the cells before IR, 10 min post IR and 24 h post IR were assessed. Representative comet images are shown in the left panel and quantifications are shown on the right. (**b**) UVRAG^FS^ inhibits UVRAG interaction with Ku70 and Ku80 and the interaction of Ku70/80 with DNA–PKcs. The 293T cells transfected with increasing amounts of Flag-UVRAG^FS^ were treated with IR (5 Gy). WCL were immunoprecipitated with anti-DNA–PKcs or anti-UVRAG, followed by immunoblotting with the indicated antibodies. (**c**) HEK293 cells stably expressing the EJ5-GFP reporter for NHEJ and the DR-GFP reporter for homologous recombination (HR) were transfected with an empty vector or Flag-UVRAG^FS^ before the induction of DSBs by *Sce*I transfection. The DNA repair activities as assessed by the reconstituted GFP signals were quantified by fluorescence-activated cell sorting. Data shown represent mean±s.d. (*n*=3). **P<0.01.

We next investigated the possible clinical relevance of UVRAG^FS^ by testing the response of CRC to 5-FU chemotherapy, the first-line treatment for CRC patients, using a tumour xenograft model. Surprisingly, UVRAG^FS^ expression significantly increased tumour sensitivity to 5-FU treatment with an approximate 10-fold reduction in tumour volumes after a 4-week administration of 5-FU (Fig. 6a), compared with a less than twofold reduction in the control group (Fig. 6a–c). Histological analyses revealed a significant reduction in cell proliferation and an increase in the number of cells undergoing apoptosis in 5-FU-treated UVRAG^FS^ tumours, in concordance with induced tumour shrinkage (Fig. 6d). In addition, UVRAG^FS^ expression in CRC cells markedly increased their sensitivity to other DNA-based cytotoxic anticancer agents, including oxaliplatin and irinotecan, as shown by reduced rates of clonogenic survival, whereas UVRAG^WT^ cells were resistant to the drugs (Fig. 6e). To examine the unexpected role of UVRAG^FS^ in tumour chemosensitivity, we measured the levels of γ-H2AX, a sensitive marker of double strand breaks (DSBs)^35^, and observed that UVRAG^FS^ SW480-tumours accumulated higher levels of γ-H2AX than the controls, which further increased with 5-FU that produces DNA strand breaks (Fig. 6d). Consistent with our observation in xenograft tumours, UVRAG^FS^ expression resulted in a significant increase of γ-H2AX foci and levels in SW480 CRC cells (Supplementary Fig. 7a,b). Furthermore, the overall levels of γ-H2AX were higher in MSI CRC cell lines expressing UVRAG^FS^ compared with the WT counterparts, and likewise, were significantly different between UVRAG^FS^-positive and -negative primary tumours (Fig. 1b,d). Adding UVRAG^WT^ to UVRAG^FS^-positive HCT116 and RKO cells at different doses clearly suppressed the levels of DSBs (Supplementary Fig. 7c), highlighting a direct involvement of UVRAG^FS^ in genetic stability. To determine whether the observed accumulation of DSB in UVRAG^FS^ cells reflects impaired DNA repair, we measured unrepaired DSBs after ionizing radiation (IR) using the comet assay. We found that IR induced comparable levels of DNA damage in vector, UVRAG^WT^ and UVRAG^FS^ cells (10 min post-IR in Fig. 7a). However, a high persistence of comet tails was observed 24 h post-irradiation in UVRAG^FS^ cells, whereas UVRAG^WT^ cells have repaired most of the damaged DNA. These data indicate that UVRAG^FS^ disrupts the rapid repair process of DSBs. The inhibitory effect of UVRAG^FS^ on DSB repair was also detected in the autophagy-competent *Atg3*^+/+^ and the autophagy-null *Atg3*^−/−^ cells (Supplementary Fig. 7d), suggesting minimal participation of autophagy in the elevated DNA damage induced by UVRAG^FS^ expression.

### UVRAG^FS^ is defective in the repair of DNA damage

We then asked whether UVRAG^FS^-associated DNA damage results from suppression of UVRAG^WT^ function, which is known to promote DSB repair by NHEJ (non-homologous end joining) through interaction with the Ku70/Ku80/DNA–PKcs complex^17^. Unlike with UVRAG^WT^, no physical interactions between UVRAG^FS^ and DNA–PK proteins could be detected (Supplementary Fig. 7e). Moreover, UVRAG^FS^ failed to translocate to sites of laser-induced DNA damage stripes containing γ-H2AX, whereas UVRAG^WT^ was enriched at the damaged sites of DSBs (Supplementary Fig. 7f). As expected, ectopic expression of UVRAG^FS^ blocked UVRAG-Ku70/Ku80 interaction, and disturbed Ku/DNA–PKcs complex formation after IR, concomitant with increased sequestration of UVRAG^WT^, again highlighting the dominant-negative effect of the FS mutation (Fig. 7b). To further establish a link between UVRAG^FS^ and the DNA-damaging phenotype observed, we evaluated the DNA repair capacity in UVRAG^FS^ cells, using a NHEJ repair reporter, the EJ5-GFP system^36^. Expression of UVRAG^FS^ alone markedly reduced the rate of NHEJ repair by over 50%, whereas it had no discernable effect on DNA homologous recombination repair (Fig. 7c). Treating cells with Nu7441, a specific inhibitor of DNA–PK^37^, abolished the effect of UVRAG^FS^ (Supplementary Fig. 7g). These results indicate that UVRAG^FS^-induced DNA damage is dependent on the inactivation of DNA-PK-mediated NHEJ, which renders tumour cells more sensitive to DNA-damaging chemotherapy.

## Discussion

Microsatellite instability as a result of MMR deficiency has been widely observed in human CRC. However, little is known of the biological consequences and pathogenic mechanisms associated with the selective gene targeting by MSI. Herein, we demonstrate that the autophagic tumour suppressor *UVRAG* represents a new *bona fide* MSI target gene in CRC and, likely, other MSI-related tumours, and that the truncating mutation in *UVRAG* enhances cellular transformation and penetrance of CRC tumour by interfering with the tumour-suppressing functions of UVRAG^WT^ in a dominant-negative manner. Furthermore, mutated UVRAG alleles sensitize CRC to DNA damage-inducing treatment, making the *UVRAG*^*FS*^ genotype a possible predictive factor for the response to chemotherapy treatment.

In this study, we found that the heterozygous deletion of the *UVRAG* A_10_ exonic DNA repeat resulted in the expression of a truncated protein using an antibody specifically recognizing UVRAG^FS^, and that it influences the expression and function of UVRAG^WT^ in a series of CRC cell lines and primary CRCs. Contrary to our findings, a previous study^7^ showed by immunoblotting that the levels of UVRAG^WT^ appeared to be unaffected by the occurrence of the UVRAG FS mutation in three MSI CRC cell lines carrying the FS mutation (HCT116, LoVo and RKO), two of which having also been used in our study (Fig. 1b). While it is difficult to explain the discrepancy between this published work and ours, it might be due to differences in experimental design and/or to different sources or passage numbers of CRC cell lines used in both studies. Nonetheless, our results are consistent with the gene expression data retrieved from a GeneChip analysis of NCI-60 cancer cell lines from TSRI (The Scripps Research Institute; data are accessible at BioGPS: http://biogps.org), correlating reduced UVRAG^WT^ expression in a subset of CRC cell lines with the *UVRAG* FS mutation. Taken as a whole, our findings and those of others suggest that inactivation of *UVRAG* is selected for during the progression of colorectal tumours, and that UVRAG^WT^ plays a suppressor role in colorectal tumorigenesis.

Previous studies have indicated that autophagy protects genomic integrity presumably by removing aged or damaged proteins and organelles^38^,^39^,^40^. We observed a significant reduction of autophagy by UVRAG^FS^ in CRC cells and primary tumours, which was even greater in the metastases. Of note, a previous study^7^ argued that UVRAG^FS^ lost Beclin1-binding activity due to the frameshift truncation. However, we found that even though UVRAG^FS^ lost more than 50% of CCD of UVRAG^WT^, it still retains a small alpha-helix structure in the CCD and remains competent for UVRAG and Beclin1 interaction, thereby neutralizing their proautophagic effect in a dose-dependent manner. However, autophagy loss could not prevent the transformed phenotype induced by UVRAG^FS^, indicative of an autophagy-independent oncogenic mechanism associated with UVRAG^FS^, as previously suggested^7^.

We found that ectopic expression of UVRAG^FS^ *per se* in both embryonic stem cells and cancer cells results in extensive centrosome amplification and concomitant aneuploidy. Indeed, this cancer-associated mutated UVRAG, which lacks CEP63-binding ability, is more than just a relic of UVRAG inactivation, it instead disturbs the association of endogenous UVRAG^WT^ with CEP63, presumably by displacing endogenous active UVRAG from the centrosome and/or by titrating out an unknown regulator into nonfunctional complexes. This is similar to what occurs with mutations in other tumour suppressors, such as p53. Certain mutated forms of p53 have not only lost their tumour-suppressive function, but have also gained a function as an oncogene^41^. Consistent with a previous study demonstrating that inappropriate microtubule nucleation due to centrosome amplification enables Rac1 activation and promotes cell invasion^31^, we found that UVRAG^FS^ promotes metastatic outgrowth and EMT properties in a Rac1-dependent manner. Our mutational and integrative analyses emphasize the critical role of UVRAG^FS^ and centrosomal stability in the context of metastatic CRC.

Despite increased oncogenic transformation, UVRAG^FS^-expressing tumours appear to be more responsive to chemotherapy that induces massive DNA damage and replication stress. Unlike UVRAG^WT^, UVRAG^FS^ cannot translocate to DSB sites and its expression further interferes with a functional complex assembly of DNA-PK, a key effector in the NHEJ pathway. As NHEJ factors are considered as genome caretakers that guarantee genomic integrity through the proper repair of DNA lesions, our data thus provide a potential mechanism by which UVRAG^FS^ elevates the levels of DNA damage via acting on NHEJ repair and sensitizes tumour cells to chemotherapy. Thus, UVRAG^FS^ may represent an important determinant in the treatment response of CRC tumours.

In summary, we have demonstrated that a cancer-derived UVRAG truncated mutant plays a role in oncogenic transformation and tumour metastasis, which explains the selection for its expression in human CRC cell lines and primary tumours with MSI. This mutant impairs UVRAG^WT^ function in autophagy and chromosomal stability. Our findings suggest that UVRAG^FS^ expression contributes to chemosensitivity through direct repression of DNA damage repair and ensuing increased cell death. This regulatory circuit may partially explain the more favourable prognosis in patients with MSI tumours than in those with MSS tumours, as previously noted^42^. It may also have potential relevance for pharmacogenetic selection of MSI cancer patients for adjuvant chemotherapy.

## Methods

### Cell culture, transfection and tumour samples

HeLa (CCL-2), 293T (CRL-3216), NIH3T3 (CRL-1658), HT29 (HTB-38), RKO (CRL-2577), LS180 (CL-187), HCT116 (CCL-247), SW480 (CCL-228) were purchased from ATCC. LIM2405, HCC2998, HCT15, COLO205, SW620, KM12 and SW48 were obtained from Dr. Guomin Li (University of Kentucky College of Medicine, USA). HeLa, 293T, immortalized MEF (iMEF), NIH3T3, HT29, RKO, LS180 and HCT116 cells were cultured in Dulbecco's modified Eagle's medium (DMEM). LIM2405, HCC2998, HCT15, COLO205, SW620 and KM12 cells were cultured in Roswell Park Memorial Institute (RPMI) 1640. SW480 and SW48 were cultured in Leibovitz’s L-15 in the absence of CO_2_. All media above were supplemented with 10% fetal bovine serum (FBS; Invitrogen), 2 mM L-glutamine, and 1% penicillin–streptomycin (Gibco-BRL). Transfections were performed with FuGENE 6 HD (Roche) or Lipofectamine 2000 (Invitrogen), following the manufacturer’s instructions. M059K and M059J cells were cultured as previously described^43^. SW480, HCT116, NIH3T3 and HeLa stable cell lines were established using a standard protocol of selection with 2 μg ml^−1^ puromycin (Sigma-Aldrich). Mouse embryonic stem cells were obtained from the Mutant Mouse Regional Resource Center, and maintained at comparable passage in GMEM (Sigma) with 15% FBS (Invitrogen), following the Mutant Mouse Regional Resource Center cell culture protocol (http://www/mmrrc.org/strains/E14/ctr_protocol.pdf). All cell lines used were mycoplasma free. Paraffin-embedded primary tumours and normal colonic tissues were obtained from patients undergoing surgery from the USC Norris Cancer Center Translational Pathology Core, CA, USA. The MSI status was determined by analyzing a comparable panel of five mononucleotide markers: NR-27, NR-21, NR-24, BAT-25 and BAT-26 (ref. 44).

### Plasmid constructs

The Flag-tagged WT UVRAG and UVRAG^FS^ mutant were constructed by cloning the cDNA of the WT and truncated UVRAG mutant into the *Afl*II/*Not*I sites of the pEF/puro-Flag vector. All constructs were confirmed by sequencing using an ABI PRISM 377 automatic DNA sequencer (Applied Biosystems).

### Mutation analysis

Genomic DNA and cDNA from cell lines and primary tumours were amplified by PCR. The primer pair (forward and reverse, respectively) is: 5′-ATGTTTTAAGCCATTATTTA-3′ and 5′-CGTTCCAGTTCATTCTG-3′. PCR products from single clones from every sample were sequenced using an automated ABI PRISM 377 automatic DNA sequencer.

### Antibodies, fluorescent dyes and other reagents

The following antibodies were used in this study: polyclonal rabbit anti-UVRAG (U7058, Sigma-Aldrich) at 1:1,000; monoclonal mouse anti-UVRAG (SAB4200005, clone UVRAG-11, Sigma-Aldrich) at 1:200; monoclonal mouse anti-Ku70 (ab-4, Thermo-fisher) at 1:5,000; monoclonal anti-Ku80 (C48E7, Cell Signaling) at 1:1,000; monoclonal mouse anti-DNA–PKcs (Ab-4, Thermo-fisher) at 1:2,000; monoclonal mouse anti-γ-tubulin (T6557, Sigma-Aldrich) at 1:2,000; Cy3 conjugated anti-γ-tubulin (C7604, Sigma-Aldrich) at 1:500; monoclonal mouse anti-α-tubulin (T6199, Sigma-Aldrich) at 1:2,000; polyclonal rabbit anti-CEP63 (16268-1-AP, ProteinTech) at 1:2,000, monoclonal mouse anti-γ-H2AX antibody (05–636, Millipore) at 1:2,000; monoclonal mouse anti-p62 at 1:3,000 (MBL, Japan); monoclonal mouse anti-LC3 at 1:500 (CAC-CTB-LC3-2-IC; clone: LC3-1703; Cosmo Bio USA); polyclonal rabbit anti-Ki67 (NB110-89719, Novus) at 1:100; monoclonal rabbit anti-cleaved caspase-3 (Asp175; #9664, Cell signaling Technology) at 1:2,000; polyclonal rabbit anti-E-cadherin (20874-1-AP, Proteintech) at 1:200; polyclonal rabbit anti-N-cadherin (PA5-29570, Pierce) at 1:1,000; monoclonal mouse anti-vimentin (MA5-11883, Pierce) at 1:1,000; monoclonal mouse anti-active Rac1-GTP (#26903, NewEast Biosciences) at 1:1,000; mouse anti-Rac-1 (#610650, BD Biosciences); monoclonal anti-Flag (F3165; clone M2; 1:2,000) were purchased from Sigma-Aldrich; The UVRAG^FS^ peptide-specific antibody was generated by immunizing rabbits with the UVRAG^FS^ peptide _234_KKKVNACS_241_, covalently coupled to keyhole limpet haemocyanin (KLH) and purchased from GenicBio. Horseradish peroxidase (HRP)-labelled or fluorescently labelled secondary antibody conjugates were purchased from Molecular Probes (Invitrogen, USA). Purified rabbit IgG as purchased from Pierce. Unless otherwise stated, all chemicals were purchased from Sigma-Aldrich.

### Immunofluorescence and confocal laser-scanning microscopy

For the centrosome-related studies, cells were fixed with cold methanol for 10 min at −20 °C, while 4% paraformaldehyde (20 min at room temperature) was used for the other studies. After fixation, cells were permeabilized with 0.2% Triton X-100 for 8 min and blocked with 10% goat serum (Gibco-BRL) for 1 h. Primary antibody staining was carried out using antiserum or purified antibody in 1% goat serum for 1–2 h at RT, or overnight at 4 °C. Cells were then extensively washed with PBS and incubated with diluted Alexa 488-, Alexa 594- and/or Alexa 633-conjugated secondary antibodies in 1% goat serum for 1 h, followed by DAPI (4′,6′-diamidino-2-phenylindole) staining. Cells were mounted using Vectashield (Vector Laboratories, Inc.). Confocal images were acquired using a Nikon Eclipse C1 laser-scanning microscope (Nikon, PA), fitted with a 60 × Nikon objective (PLAPO, 1.4 NA), and Nikon image software. Images were collected at 512 × 512-pixel resolution. The stained cells were optically sectioned in the *z* axis. For multichannel imaging, fluorescent staining was imaged sequentially in line-interlace modes to eliminate crosstalk between the channels. The step size in the *z* axis varied from 0.2–0.5 mm to obtain 16 slices per imaged file. All experiments were independently repeated several times. The investigators conducted blind counting for quantification. Values indicate the mean±s.d. of at least three independent experiments.

### Histopathology and immunohistochemistry

Tissue sections from the indicated mouse models were fixed in 10% buffered formalin and embedded in paraffin. Tissue sections were routinely stained with haematoxylin and eosin. For immunohistochemistry staining, tissue slides were deparaffinized in xylene and rehydrated in alcohol. Endogenous peroxidase was blocked with 3% hydrogen peroxide. Antigen retrieval was achieved using a microwave and 10-mM citric sodium buffer (pH 6.0). Sections were then incubated overnight at 4 °C with the primary antibody. Antibody binding was detected with Envision Dual Link System-HRP DAB kit (K4065, Dako). Sections were then counterstained with haematoxylin. For negative control, the primary antibody was replaced with the buffer. The mitotic index was quantified by viewing and photographing 10 random high-power field of each tissue section on a Nikon microscope, using a 40 × objective. For evaluation and scoring of immunohistochemical data, we randomly selected 10 fields within the tumour area under high-power magnification (× 400) for evaluation. The investigators conducted blind counting for each quantification-related study.

### Immunoblotting and immunoprecipitation

For immunoblotting, polypeptides were resolved by SDS–PAGE and transferred to a PVDF membrane (Bio-Rad). Membranes were blocked with 5% non-fat dry milk, and probed with the indicated antibodies. HRP-conjugated goat secondary antibodies were used (1:10,000, Invitrogen). Immunodetection was achieved with the Hyglo chemiluminescence reagent (Denville Scientific), and detected by a Fuji ECL machine (LAS-3000). For co-immunoprecipitation, cells were lysed in 1% NP40 lysis buffer (25 mM Tris pH 7.5; 300 mM NaCl, 1 mM EDTA, 1% NP40), supplemented with a complete protease inhibitor cocktail (Roche). After preclearing with protein A/G agarose beads for 1 hr at 4 °C, whole-cell lysates were used for immunoprecipitation with the indicated antibodies. Generally, 1–4 μg commercial antibody was added to cell lysate, which was incubated at 4 °C for 8–12 h. After addition of protein A/G agarose beads, incubation was continued for another 2 h. Immunoprecipitates were extensively washed with NP40 lysis buffer and eluted with SDS–PAGE loading buffer by boiling for 5 min before resolution by SDS–PAGE.

### Soft agar anchorage-independent growth assay

To evaluate anchorage-independent colony formation, engineered cells (10^4^) were suspended in complete medium containing 0.3% Nobel agar (Difco) supplemented with 2 μg ml^−1^ puromycin and plated in 6-well plates over a basal layer of 0.5% agar in complete medium. Colonies were scored 21 days after plating and were photographed by phase-contrast microscopy. Images were captured with the QCapture software program. Clonogenicity was determined in triplicate experiments.

### *In vitro* wound-healing assay

The cell invasive activity was determined using the wound-healing assay^45^. Briefly, cells (2.5 × 10^5^) were seeded in 12-well slide chambers and grown into a 100% confluent monolayer culture. The confluent cell monolayer was scratched with a pipette tip, followed by media replacement. After 24 h, the width of the mean wound distance was calculated using software connected to Nikon Eclipse digital inverted microscope. To evaluate the ‘wound closure’, 10 randomly selected points along each wound were marked, and the horizontal distance the migrating cells travelled into the wound was measured.

### *In vitro* cell migration assays

A Transwell system (Corning, NY, USA) was used to evaluate cell migration. The upper and lower chambers were separated by a polycarbonate membrane with pores of 8-μm coated with fibronectin (BD Biosciences, CA, USA) on the lower surface. Cells (2 × 10^5^) suspended in 100 μl serum-free medium were seeded onto the upper chamber, and 800 μl of medium with 10% FBS was added to the lower chamber. After 24-h incubation at 37 °C with 5% CO_2_, the medium was removed from the upper chamber. The non-invading cells on the upper side of the chamber were scraped off with a cotton swab. Cells on the bottom side of the membrane were fixed, stained with crystal violet and mounted. The migration activity of cancer cells was determined by counting cells in 10 different viewing fields using a microscope at × 200 magnification. Each assay was repeated three times.

### Clonogenic cell survival assay

The log-phased cells were plated in six-well plates overnight, allowing cells to attach to the plates. After chemotherapy drug treatment (24 h exposure), cells were trypsinized, counted and replated at appropriate dilutions for colony formation. After 10–14 days of incubation, colonies were fixed with methanol/acetic acid (3:1), stained with crystal violet and counted. Plating efficiency was determined for each individual cell line^46^ and the surviving fraction (SF) was calculated based on the number of colonies that arose after treatment, expressed in terms of plating efficiency. Each experiment was repeated three times.

### *In vivo* tumorigenicity assay

To measure *in vivo* tumorigenicity, engineered NIH3T3 and SW480 cells expressing WT or the mutant form of UVRAG (5 × 10^6^) were transplanted into the flanks of six-week-old female NCR nude mice (Charles River). Ten mice per cell line were used. Mice were monitored triweekly for the development of tumours, and necropsied after a 3-week observation period. The tumour growth was monitored by measurements of tumour length (L) and width (W) and tumour volume was calculated^47^ using the following formula: Volume=4/3 × *π* × (1/2 width)^2^ × 1/2 length. All animal studies were performed in compliance with the University of Southern California Institutional Animal Care and Use Committee guidelines.

### *In vivo* metastasis assay

A midline incision was made on the left flank, and the spleen was exteriorized. SW480 cells (10^6^ cells) were injected into the spleen, after which the wound was closed with surgical metal clips. The mice were sacrificed after 8 weeks, and their spleen, liver, lungs and lymph nodes were removed and examined for tumour metastases. The organ specimens were formalin-fixed and paraffin-embedded for histological analysis. Alternatively, GFP-labelled cells can be tracked using bioluminescence imaging at the end of experiment. Briefly, mice were placed in the induction chamber with 2% isoflurane in oxygen. GFP activity was localized and quantified using an IVIS 200 image system. Images were taken with an excitation wavelength of 465 and emission wavelength ranging from 500 to 540. Imaging processing and analysis, including flat fielding, adaptive background subtraction and spectral unmixing were performed with Living Image 3.0 software.

### Autophagy analyses

Quantitative GFP–LC3 light microscopy assay was performed in NIH3T3 cells expressing the WT or FS mutant of UVRAG, then transfected with a GFP–LC3-expressing plasmid^24^. Autophagy was then induced by 100 nM rapamycin (Sigma-Aldrich) for 2–6 h in DMEM containing 1% FBS. For autophagic flux, the rapamycin-treated cells were cultured in DMEM containing 100 nM Bafilomycin A_1_ for 2 h. LC3 mobility shift and levels were detected by immunoblotting^12^,^48^.

### Neutral comet assay

Neutral comet assay was performed using the CometAssay kit (Trevigen) following the manufacturer’s instruction. Briefly, 10 μl of cell suspension (10^5^ cells per ml) was carefully mixed with 90 μl of molten LMAgarose. After solidification, slides were immersed in Lysis Solution at 4 °C for 1 h, and equilibrated in chilled neutral electrophoresis buffer for 30 min. Electrophoresis was performed in neutral electrophoresis buffer for 1 h with an electric field of 1 volt cm^−1^. Slides were further treated with DNA Precipitation Solution, followed by 70% ethanol for 30 min each at room temperature. After air drying, cells were stained with SYBR Green (1 μg ml^−1^) or Propidium Iodide (1 μg ml^−1^). Comet images were captured using an epifluorescence microscope (Nikon Eclipse C1). To analyse the images, cells were scored into three categories based on tail length (no tails, tail length shorter than 20 μm, tail length longer than 20 μm), and quantified.

### Laser microirradiation

Laser microirradiation was done essentially as described before^49^. Cells grown on coverslips were incubated for 24 h in medium containing 10 μM BrdU (Sigma-Aldrich). Laser microirradiation was carried out with a Nikon C1 confocal microscope (Nikon) equipped with a 37 °C CO_2_ chamber and a diode laser (Melles Griot). DSBs restricted to the laser path were generated across the nuclei in 50 cells per coverslip, using the 100 × oil objective and 30% of laser power (*λ*=405 nm) for 100 scans. Cells were then returned to tissue culture incubator at 37 °C, fixed 1 h later and analysed by immunofluorescence as described below. Laser-induced DNA damage was visualized with the γ-H2AX antibody (Millipore) and the UVRAG antibody (Sigma). Images were taken with a Nikon C1 confocal microscope (Nikon) and Axio Imager 2 (Zeiss).

### *In vivo* DNA DSB Repair

To measure the DNA DSB repair activity, a GFP-based chromosomally integrated reporter was utilized^50^. In brief, the HEK293 cells stably expressing EJ5-GFP reporter were transfected with empty vector or UVRAG^FS^ plasmid. Two days later, a secondary transfection was performed with the same plasmids plus an I-*Sce*I expression vector (pCBASce), together with pmCherry as a transfection indicator. Cells were collected after another 48 h, and analysed by standard flow cytometry. UVRAG expression was verified by western blotting. The repair activity of DSB generated by I-*Sce*I was calculated by the percentage of GFP-positive (repaired) cells in the mCherry-positive cells (transfected).

### Chromosomal analysis by SKY

SKY analysis of embryonic stem cells was performed. Briefly, metaphase chromosome were prepared from exponentially growing cells after treatment with colcemid (KaryoMAX, GIBCO) at 0.1 μg ml^−1^ for 1 hr (ref. 51). Cells were swollen in prewarmed 0.56% KCl for 10 min at 37 °C, then carefully fixed in methanol:acetic acid (3:1) overnight and kept at −20 °C. Metaphase spreads were prepared by dropping cells in the fixative onto chilled Superfrost glass slides (Fisher Scientific) at 25 °C and 60% of humidity. After air drying and pepsin digestion, slides were denatured at 80 °C for 5 min, hybridization was performed using SKY probe (Applied Spectral Imaging, San Diego) and fluorescence-conjugated secondary antibodies in accordance with the manufacturer’s specification. Metaphase images were captured and analysed using a SpectraCube imaging system and software (Applied Spectral Imaging). At least 20 metaphases from each cell line were scored for chromosomal aberration.

### Genomic analysis of publically available datasets

All data for UVRAG frameshift mutation in human CRC, gastric, and endometrial cancers with MSI were obtained from SelTarbase (http://www.seltarbase.org/) and primary public sources^6^,^52^,^53^,^54^. All data for DNA sequence alteration, chromosomal structure variants, and clinical information in gastric cancer were obtained from cBioportal (http://www.cbioportal.org)^55^,^56^ and primary sources^57^. All statistical analyses were carried out using the *R* software package. Circos plots were carried out using Circos (http://circos.ca/).

### Statistical analysis

All experiments were independently repeated at least three times. To ensure adequate power and decrease estimation error, we used large sample sizes and multiple independent repeats by independent investigators. Multiple lines of experiments including different quantification methods were used for the consistent and mutually supportive results. The sample size was chosen according to the well-established rule in the literature as well as our ample experience in previous research. Data are presented as the mean±s.d. Statistical significance was calculated using the Student’s *t*-test or one-way analysis of variance test using GraphPad Prism 5.0 (GraphPad Software, Inc.), unless otherwise stated. A *P* value of ≤0.05 was considered statistically significant^58^.

## Additional information

**How to cite this article:** He, S. *et al*. Truncating mutation in the autophagy gene *UVRAG* confers oncogenic properties and chemosensitivity in colorectal cancers. *Nat. Commun*. 6:7430 doi: 10.1038/ncomms8430 (2015).

## Supplementary Material

Supplementary Information Supplementary Figures 1-10, Supplementary Table 1 and Supplementary Reference
