# Supplementary material for: Truncating mutation in the autophagy gene UVRAG confers oncogenic properties and chemosensitivity in colorectal cancers
Source: Nat Commun. 2015 Aug 3;6:7839. doi: 10.1038/ncomms8839 (PMC4526116; doi:10.1038/ncomms8839)
Supplement: Supplementary Information — Supplementary Figures 1-10, Supplementary Table 1 and Supplementary Reference [file ncomms8839-s1.pdf]

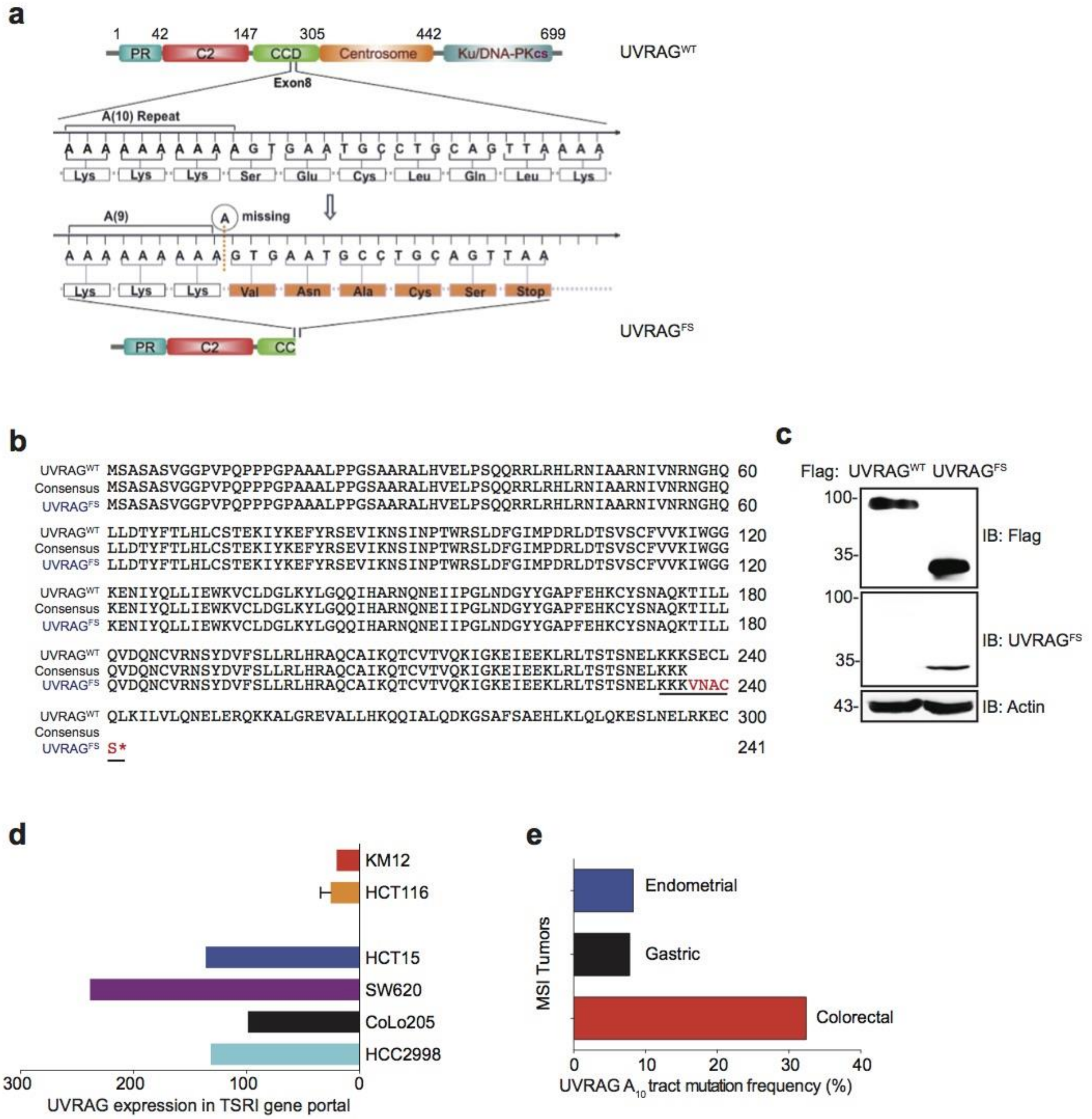

Supplementary Fig. 1

Supplementary Figure 1. Frameshift (FS) mutation in *UVRAG*.

(**a**) Schematic diagram of the FS mutation of UVRAG in exon 8 containing the highly instable A_{10} DNA repeat, generating a premature stop codon (TAA) and thereby a truncated protein ($UVRAG^{FS}$). Domain organization of wild-type UVRAG and amino acid compositions shown at

the top. PR, proline rich; CCD, coiled-coil domain; Ku/DNA-PKcs, Ku70/Ku80/DNA-dependent protein kinase catalytic subunit.

(**b**) The full-length amino acid sequence of UVRAG^{FS} compared to UVRAG^{WT}. The truncation is due to the frameshift mutation in exon 8. Amino acid alterations were highlighted in red. *, stop codon.

(**c**) Detection of the FS mutant of UVRAG *in vivo* with UVRAG FS-peptide-derived antibody. HEK293T cells were transfected with Flag-UVRAG^{WT} and Flag-UVRAG^{FS}. Whole cell lysates (WCL) were immunoblotted (IB) with anti-Flag and the antibody against UVRAG^{FS}. Note that UVRAG^{FS}-specific antibody does not recognize WT UVRAG.

(**d**) UVRAG expression in six different CRC cell lines of the NCI-60 panel from TSRI (The Scripps Research Institute). TSRI Data are accessible at BioGPS: http://biogps.org).

(**e**) Distribution of the FS mutation at the A_{10} repeats in *UVRAG* in human cancers with MSI (see Seltarbase; accessible at http://www.seltarbase.org).

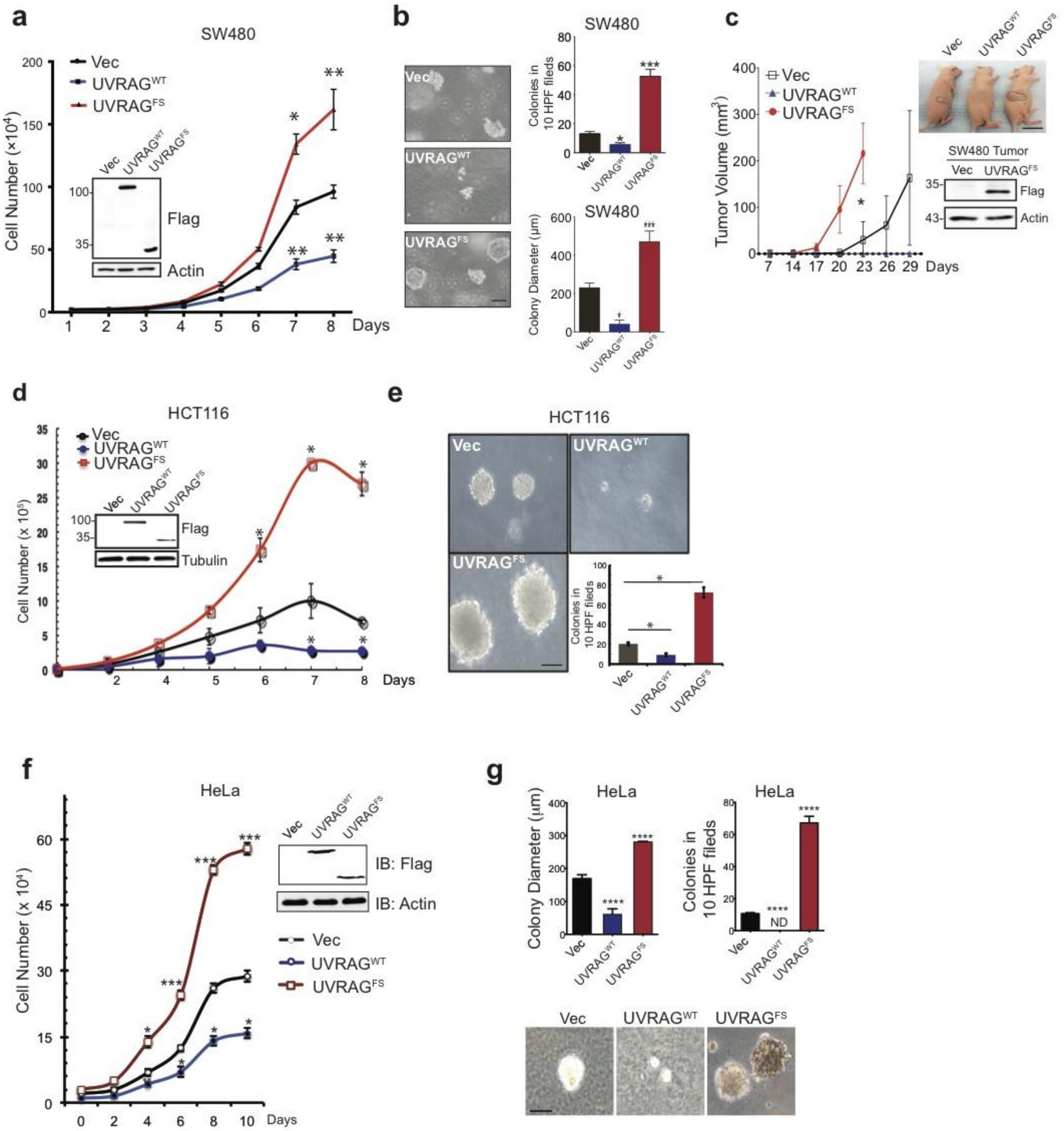

Supplementary Fig. 2

Supplementary Figure 2. Oncogenic function of UVRAG^{FS}.

(**a, b, c**) UVRAG^{FS} promotes cell proliferation (**a**), anchorage-independent colony formation (**b**), and subcutaneous tumor formation (**c**) in a mouse xenograft model with MSS SW480 cells. SW480 cells stably expressing empty vector, UVRAG^{WT}, or UVRAG^{FS} were seeded at 5000

cells per well in the present of puromycin (2 μg/ml) and counted over time in triplicate (a). Values are the means ± SD (n = 3). Expression of WT and UVRAG^{FS} was detected by western blot with actin serving as a loading control. SW480 cells in (**a**) were seeded (5 x 10^4 cells per plate) in soft agar, cultured for two weeks, fixed, and stained with crystal violet (**b**). Colony numbers and diameters were evaluated from three independent experiments, with 10 random fields per condition. Values are the means ± SD (n = 3). For tumor growth *in vivo* **(c)**, SW480 cells (10^6) as described in (**a**) were injected subcutaneously into nude mice. Tumor volumes were measured daily over time and representative images of mice with colon tumors are shown. WCL of crude tumor tissues from vector and UVRAG^{FS} mice were subjected to western blot analysis using Flag and actin antibodies. Data shown are representative of three independent analyses of randomly selected tumor species from mice. Scale bar, 5 mm.

(**d, e**) UVRAG^{FS} promotes cell proliferation (**d**) and anchorage-independent colony formation (**e**) of MSI HCT116 cells. HCT116 cells stably expressing empty vector, UVRAG^{WT}, or UVRAG^{FS} were seeded at 5000 cells per well and counted over time in triplicates. Values are the means ± SD (n = 3). WCL were blotted with Flag and tubulin antibodies (**d**). Alternatively, HCT116 cells were seeded in soft agar and colony numbers were evaluated as described above. Values are the the means ± SD (n = 3).

(**f, g**) UVRAG^{FS} promotes cell proliferation (**f**) and anchorage-independent colony formation (**g**) of HeLa cells. HeLa cells stably expressing empty vector, UVRAG^{WT}, or UVRAG^{FS} were seeded and counted over time in triplicate as described above, and lysates were blotted with Flag and actin antibodies (**f**). Additionally, HeLa cells were plated in soft agar and colonies counted as described above. All values are the means ± SD (n = 3).

Scale bars, 50 μm. *, $P < 0.05$; **, $P < 0.01$; ***, $P < 0.001$.

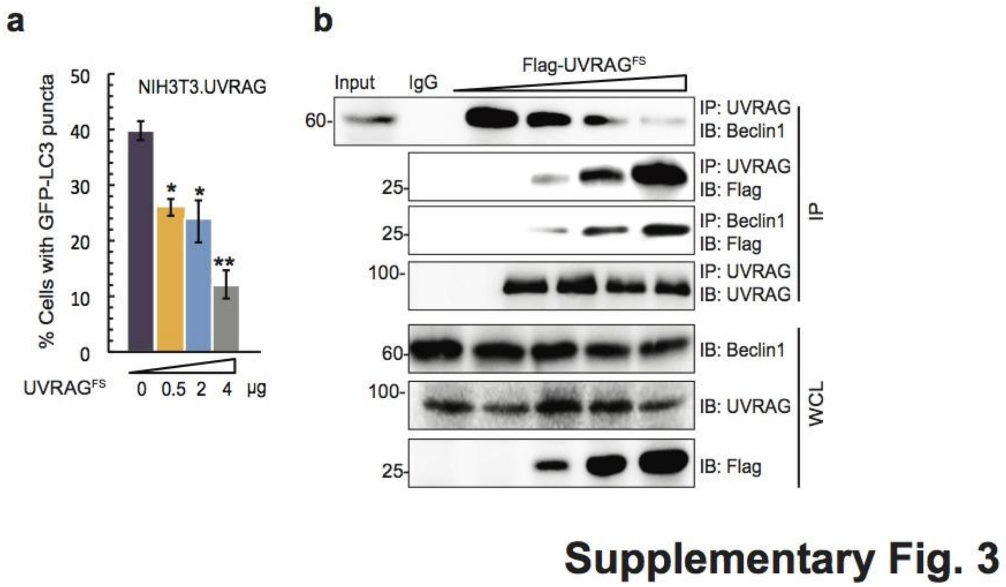

Supplementary Figure 3 Inhibitory effect of UVRAG^{FS} on autophagy.

(**a**) Dominant negative inhibition of autophagy by UVRAG^{FS}. NIH3T3.UVRAG cells were transfected with increasing amount of UVRAG^{FS} (0, 0.5, 2, 4 μg) together with GFP-LC3 and treated with rapamycin. The percentage of cells with GFP-LC3 puncta was quantified. Data represent mean ± SD (n = 3). *, $P < 0.05$; **, $P < 0.01$.

(**b**) UVRAG^{FS} inhibits the interaction between endogenous UVRAG and Beclin1. HEK293T cells were transfected with increasing amounts of Flag-UVRAG^{FS}. WCL were used for co-immunoprecipitation (co-IP) with anti-UVRAG, anti-Beclin1, or anti-Flag, followed by IB with the indicated antibodies.

a

UVRAG +/+ Complement with Vector	Chromosome Number	Numerical Defect	Structual Defect
Vecotr-01	40	-	-
Vecotr-02	40	-	-
Vecotr-03	40	-	-
Vecotr-04	40	Loss (-18); Gain(+15)	-
Vecotr-05	40	-	-
Vecotr-06	40	-	-
Vecotr-07	40	-	-
Vecotr-08	35	Loss (-5.-9,-11,-14,-18)	
Vecotr-09	40	-	-
Vecotr-10	40	-	-
Vecotr-11	40	-	-
Vecotr-12	40	-	-
Vecotr-13	40		-
Vecotr-14	40	-	-
Vecotr-15	40	-	-
Vecotr-16	40	-	-
Vecotr-17	38	Loss (-3,-19)	Del(1)
Vecotr-18	40	-	-
Vecotr-19	35	Loss (-4,-8,-10,-14,-15)	-
Vecotr-20	39	Loss(-3)	Del(14)

UVRAG +/+ Complement with $UVRAG^{FS}$	Chromosome Number	Numerical Defect	Structual Defect
FS-01	40	-	-
FS-02	38	Loss (-12,-17)	-
FS-03	35	Loss (-Y,-7,-13,-14,-17)	-
FS-04	40	-	-
FS-05	40	-	-
FS-06	30	Loss (-Y,-1,-3,-4,-10 15,-17,-19) Gain(+14)	-
FS-07	40	-	-
FS-08	30	Loss (-X,-Y,-3,-11,-12,-13,-14,-17,-17,-18)	-
FS-09	25	Loss (-Y,-1,-7,-9,-10,-10,-11,-11,-12,-15,-16,-16,-17,-17,-18)	-
FS-10	40	-	-
FS-11	40	-	-
FS-12	34	Loss (-X,-1,-11,-11,-12,-15)	-
FS-13	25	Loss (-X,-Y,-1,-3,-7,-9,-9,-11,-11,-12,-13,-14,-16,-17,-19)	-
FS-14	40	-	-
FS-15	40	-	-
FS-16	40	-	-
FS-17	40	-	Del(3)
FS-18	37	Loss (-6,-11,-16)	-
FS-19	39	Loss (-11,-19); Gain(+2)	-
FS-20	40	-	-

b

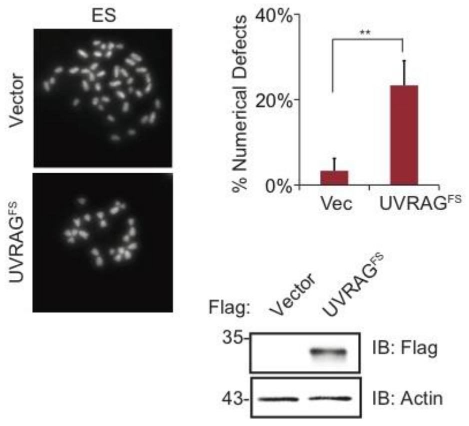

c

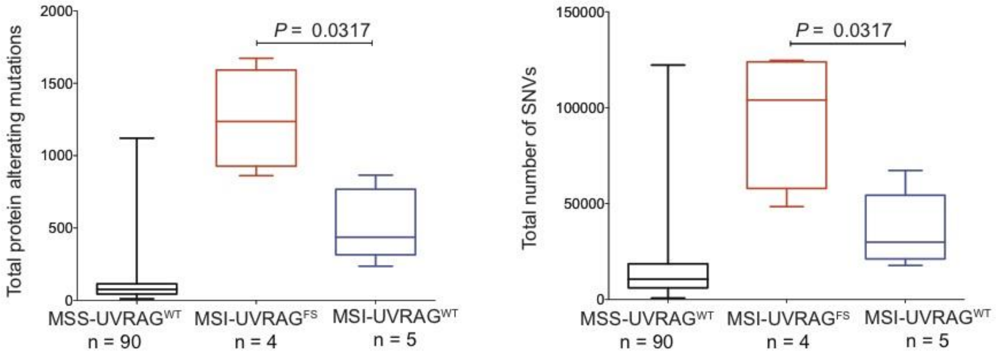

d

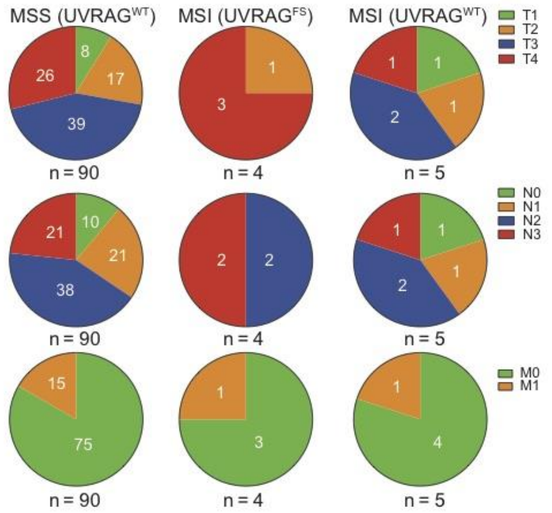

Supplementary Fig. 4

Supplementary Figure 4. UVRAG^{FS} induces chromosomal aneuploidy.

(**a**) Karyotypes of 20 metaphases from vector and UVRAG^{FS} ES cell lines are listed. Del, deletion.

(**b**) Representative images of metaphase spread from ES cells stably expressing an empty vector or Flag-UVRAG^{FS} (DAPI stained). The percentage of cells with abnormal karyotype (aneuploidy) was quantified (top right) (**, $P < 0.01$, from three independent experiments). The expression of UVRAG^{FS} was analyzed by immunoblot with anti-Flag and actin antibodies (bottom right).

(**c**) Boxplot representation of total protein-altering mutations (left) and total number of single nucleotide variants (SNVs, right) in MSS (n = 90), UVRAG^{WT}-MSI (n = 4) and UVRAG^{FS}-MSI (n = 5) gastric cancer of the Pfizer & UHK cohort[1]. The Mann-Whitney test was used.

(**d**) UVRAG^{FS} expression is associated with advanced TNM stage in the Pfizer & UHK cohort[1]. TNM, Tumor, Node, Metastasis.

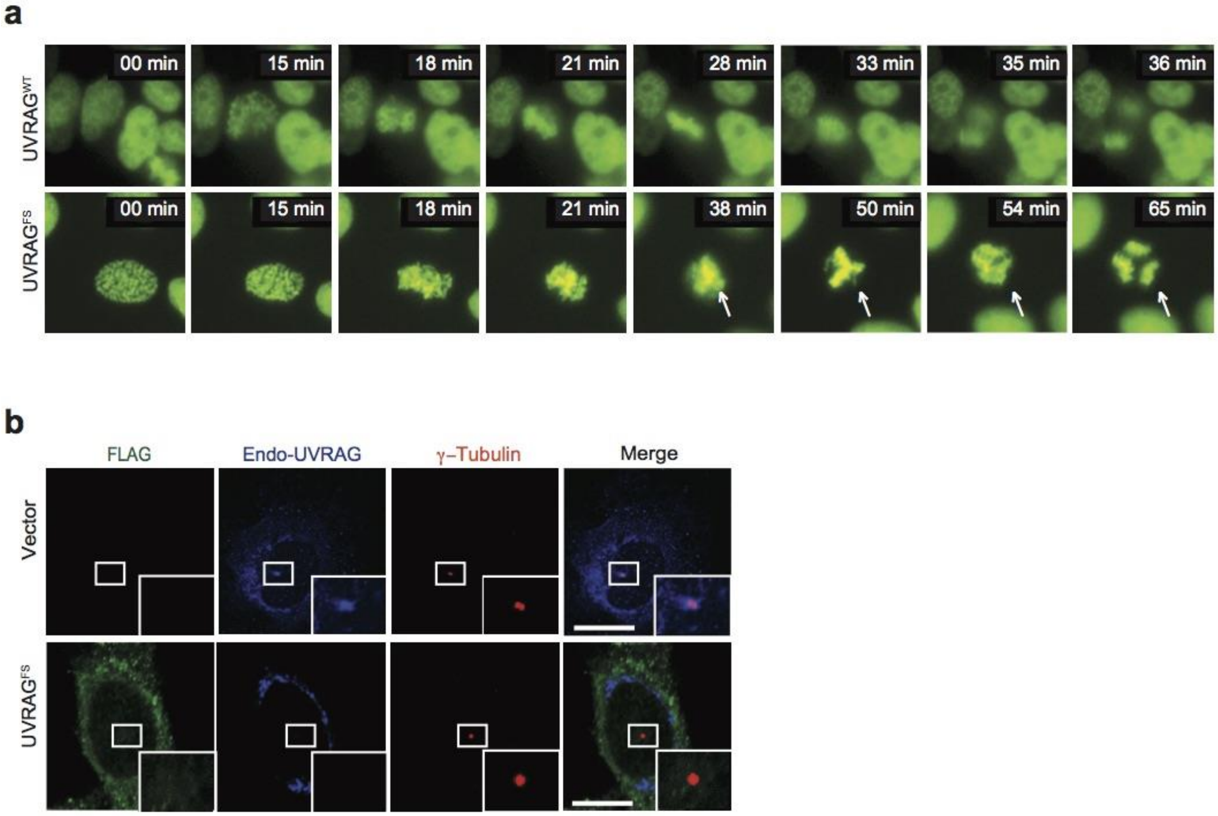

Supplementary Fig. 5

Supplementary Figure 5. UVRAG^{FS} induces centrosome instability and chromosomal missegregation.

(**a**) Time lapse of cell division in UVRAG^{WT} and UVRAG^{FS} SW480 cells. Arrows denote malformed spindles and prolonged mitosis upon UVRAG^{FS} expression.

(**b**) Dislocalization of endogenous UVRAG from the centrosome structure by UVRAG^{FS}. HeLa cells stably expressing vector or Flag-UVRAG^{FS} were immunostained with anti-Flag (green), anti-UVRAG (blue) that does not recognize UVRAG^{FS}, and γ-Tubulin. Note that UVRAG no longer localizes to the centrosomes in UVRAG^{FS}-expressing cells, in contrast to control cells where UVRAG is distinctly associated with the centrosome. Scale bars, 10 μm.

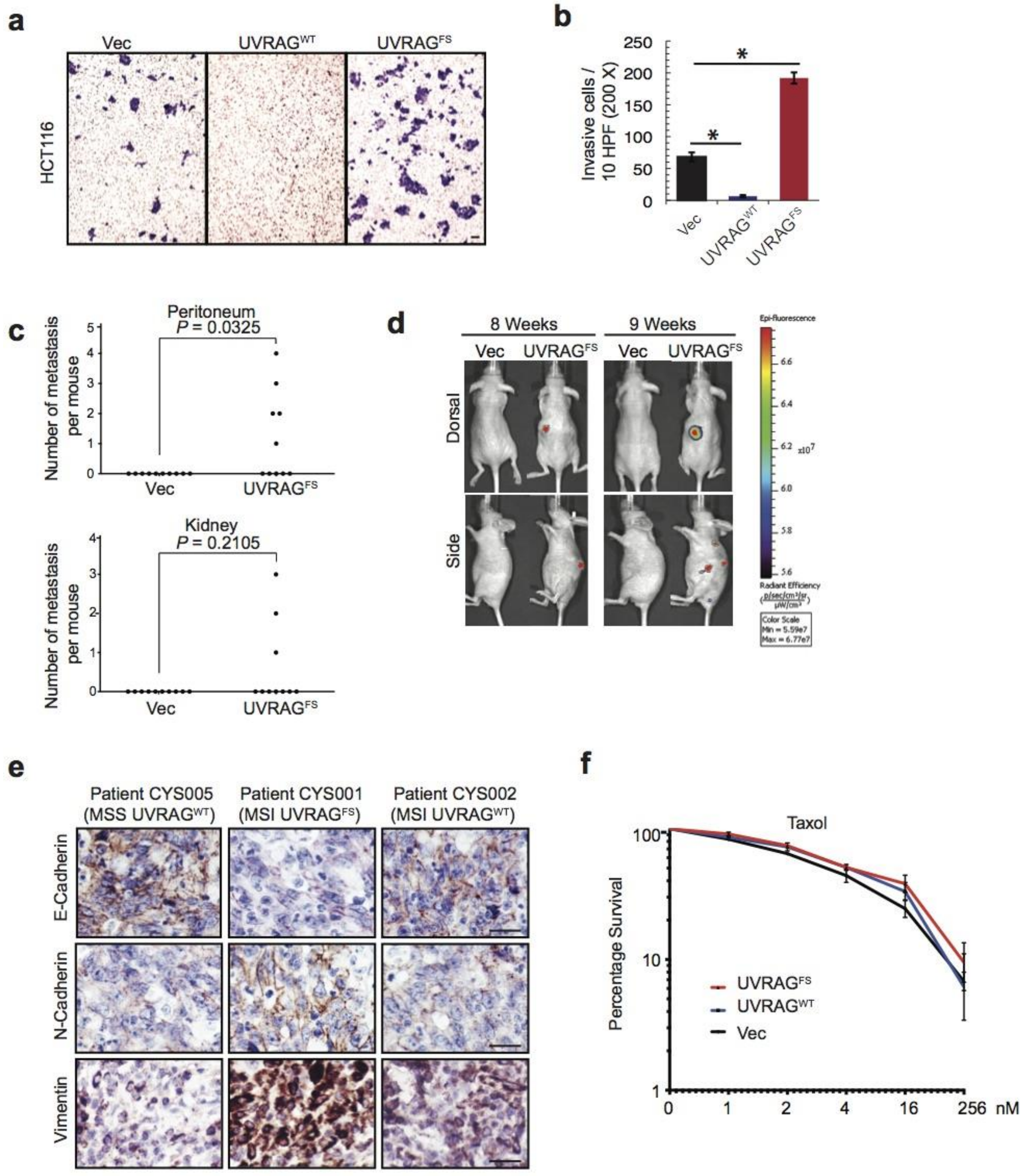

Supplementary Fig. 6

Supplementary Figure 6. UVRAG^{FS} enhances tumor metastasis and EMT change.

(**a, b**) UVRAG^{FS} promotes cell migration *in vitro*. Migration of HCT116 cells stably expressing vector, UVRAG^{WT}, or UVRAG^{FS} was measured by the trans-well migration assay. Data are

presented as mean ± SD (n = 3). Images were captured at 72 hr under 60x magnification (**a**). Scale bar, 10 μm. Invasive cells per 10 HPF were quantified (**b)**.

(**c**) Quantification of tumor metastasis in the peritoneum and kidney of mice that were inoculated by intrasplenic injection with SW480 cells stably expressing Vec and $UVRAG^{FS}$. Fisher's exact test was used for statistical analysis.

(**d**) Dorsal and lateral bioluminescent images of metastatic lesions of GFP-expressing SW480 cells in live nude mice at the indicated time after splenic transplantation of SW480.Vec or SW480.$UVRAG^{FS}$ cells. Radiant efficiency is expressed as p/sec/cm^2/sr/(μW/cm^2).

(**e**) Immunohistochemical analysis of EMT-related protein expression in human primary CRC specimen with $UVRAG^{WT}$ or $UVRAG^{FS}$ molecular subtype obtained from three representative patients.

(**f**) SW480.Vec, SW480.$UVRAG^{WT}$, and SW480.$UVRAG^{FS}$ cells were treated with Taxol, followed by colony survival assay. Data are mean ± SD (n = 3). No significant difference was detected.

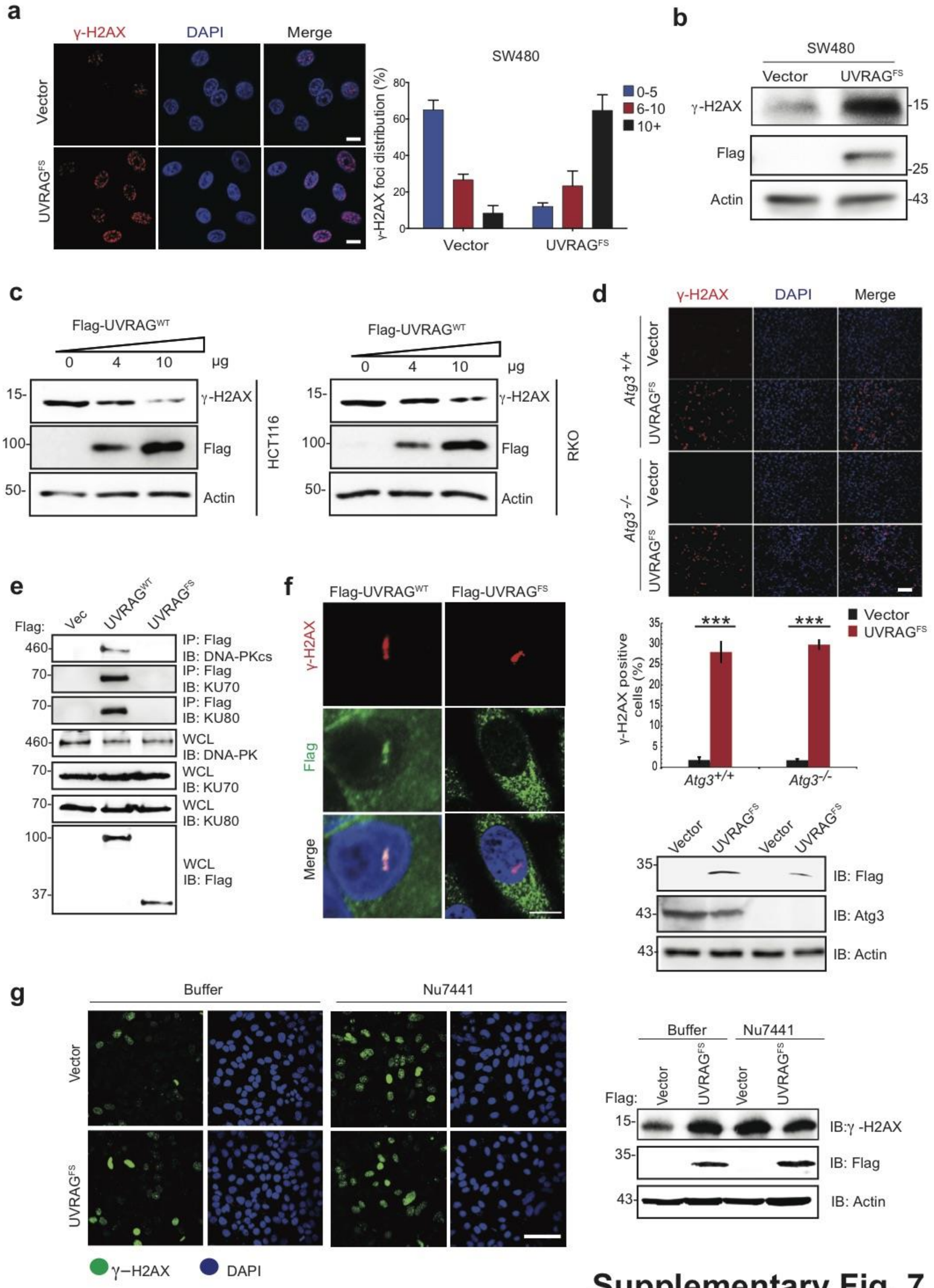

Supplementary Fig. 7

Supplementary Figure 7. UVRAG^{FS} impairs DNA double-strand-break (DSB) repair.

(**a, b**) UVRAG^{FS} expression leads to the accumulation of DNA DSBs. SW480.Vec and SW480. UVRAG^{FS} cells were stained with anti-γ-H2AX (red) and DAPI (blue). Representative images of γ-H2AX foci are shown (left). The percent distribution of cells with different γ-H2AX foci is determined (right). Western blot shows the levels of endogenous γ-H2AX and UVRAG^{FS} expression in these cells (**b**). Data are the means ± SD (n = 3). Scale bar, 5 μm.

(**c**) UVRAG^{FS}-associated accumulation of DNA DSBs can be antagonized by exogenous UVRAG^{WT} dose-dependently. HCT116 (left) and RKO (right) cells were transfected with increasing amount of Flag-UVRAG^{WT}. The levels of endogenous γ-H2AX expression in these cells were examined by western blot. Actin serves as a loading control.

(**d**) UVRAG^{FS}–associated accumulation of DNA DSBs is independent of autophagy. *Atg3*$^{+/+}$ and *Atg3*$^{-/-}$ iMEF cells stably expressing Vec and Flag-UVRAG^{FS} were stained with anti-γ-H2AX (red) and DAPI (blue). Representative images of γ-H2AX foci are shown (up). The percentage of cells with γ-H2AX foci is determined (middle) and immunobloting shows the expression of Flag, Atg3 and actin in these cells (bottom). Scale bar, 50 μm. ***, $P < 0.001$.

(**e**) UVRAG^{FS} does not interact with the DNA-PK complex. WCL of 293T cells transfected with vector control, Flag-UVRAG^{WT}, or UVRAG^{FS} were immunoprecipitated with anti-Flag, followed by IB with the indicated antibodies. The bottom four panels show input protein expression.

(**f**) UVRAG^{FS} fails to be recruited to laser-induced DNA damage stripes. SW480.UVRAG^{WT} and SW480.UVRAG^{FS} cells were subject to focal laser microirradiation followed by immunofluorescence (1 hr after damage) with the indicated antibodies against γ-H2AX,

UVRAG, and DAPI (for nuclei). Note that unlike $UVRAG^{WT}$ (left panel), $UVRAG^{FS}$ (right panel) is defective in the recruitment to the γ-H2AX-positive DSBs sites. Scale bar, 10 μm.

(**g**) $UVRAG^{FS}$ inhibits DSBs repair through DNA-PK. MEF cells expressing vector or $UVRAG^{FS}$ were treated with buffer vehicle or Nu7441 (50 nM). DSBs were measured by immunostaining of γ-H2AX (green). Immunoblotting shows the levels of endogenous γ-H2AX and $UVRAG^{FS}$ in these cells (right). Scale bar, 50 μm.

Fig. 1

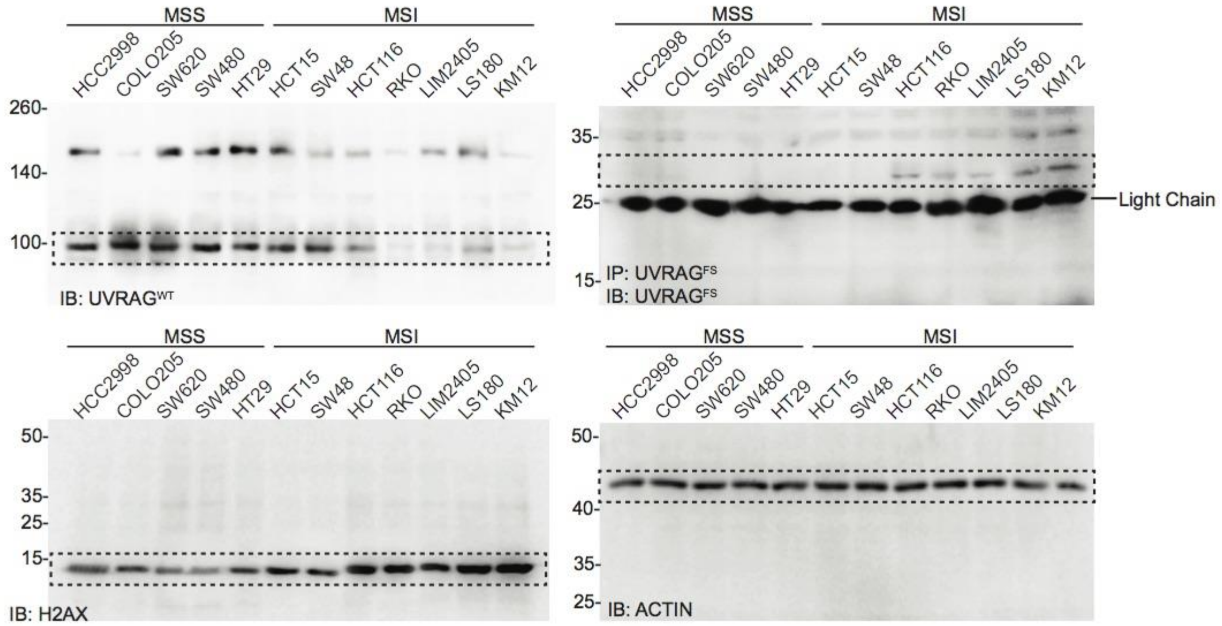

Fig. 3b

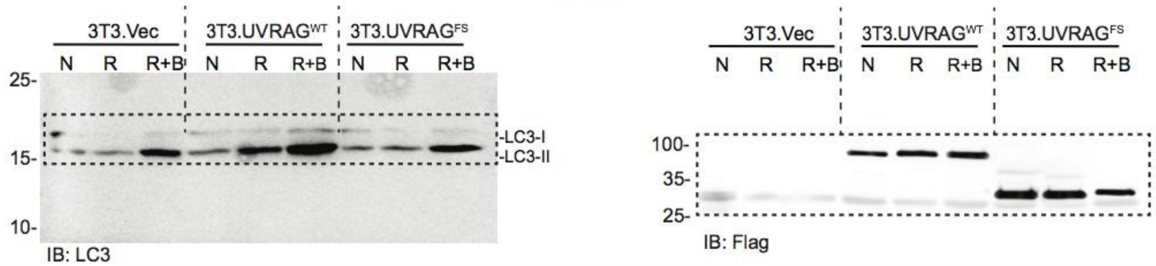

Fig. 3d

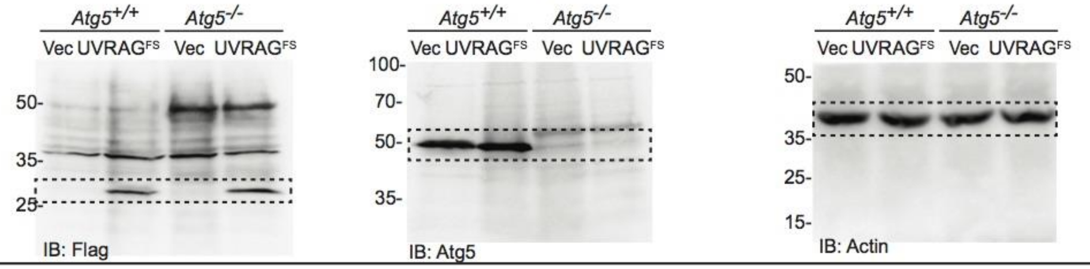

Fig. 4e

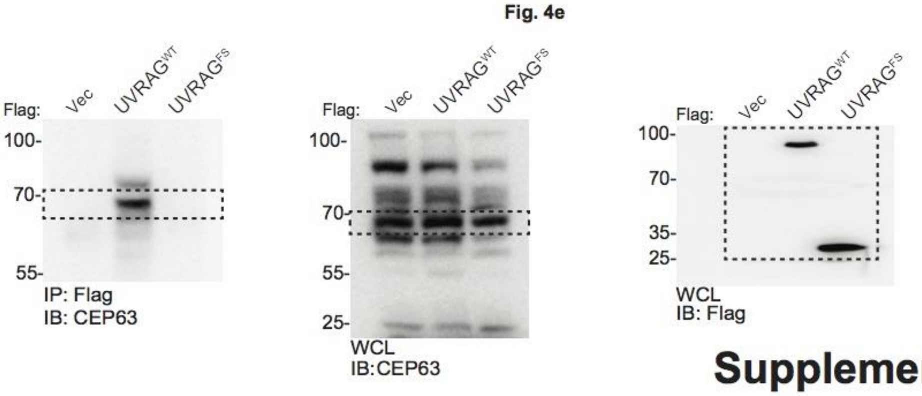

Supplementary Fig. 8

Supplementary Figure 8. The uncropped blots showing the experiments displayed in the main and supplementary figures

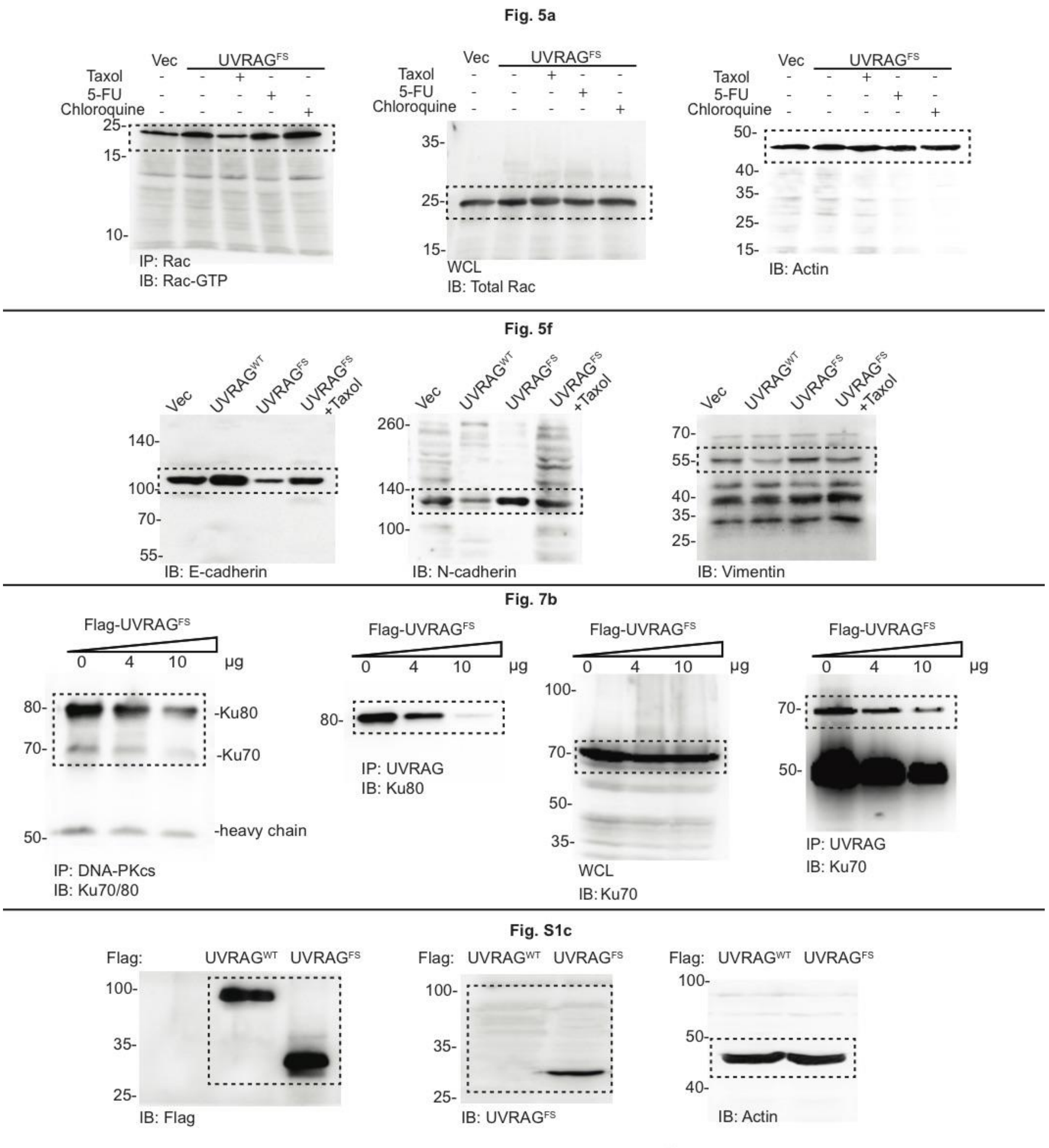

Supplementary Fig. 9

Supplementary Figure 9. The uncropped blots showing the experiments displayed in the main and supplementary figures

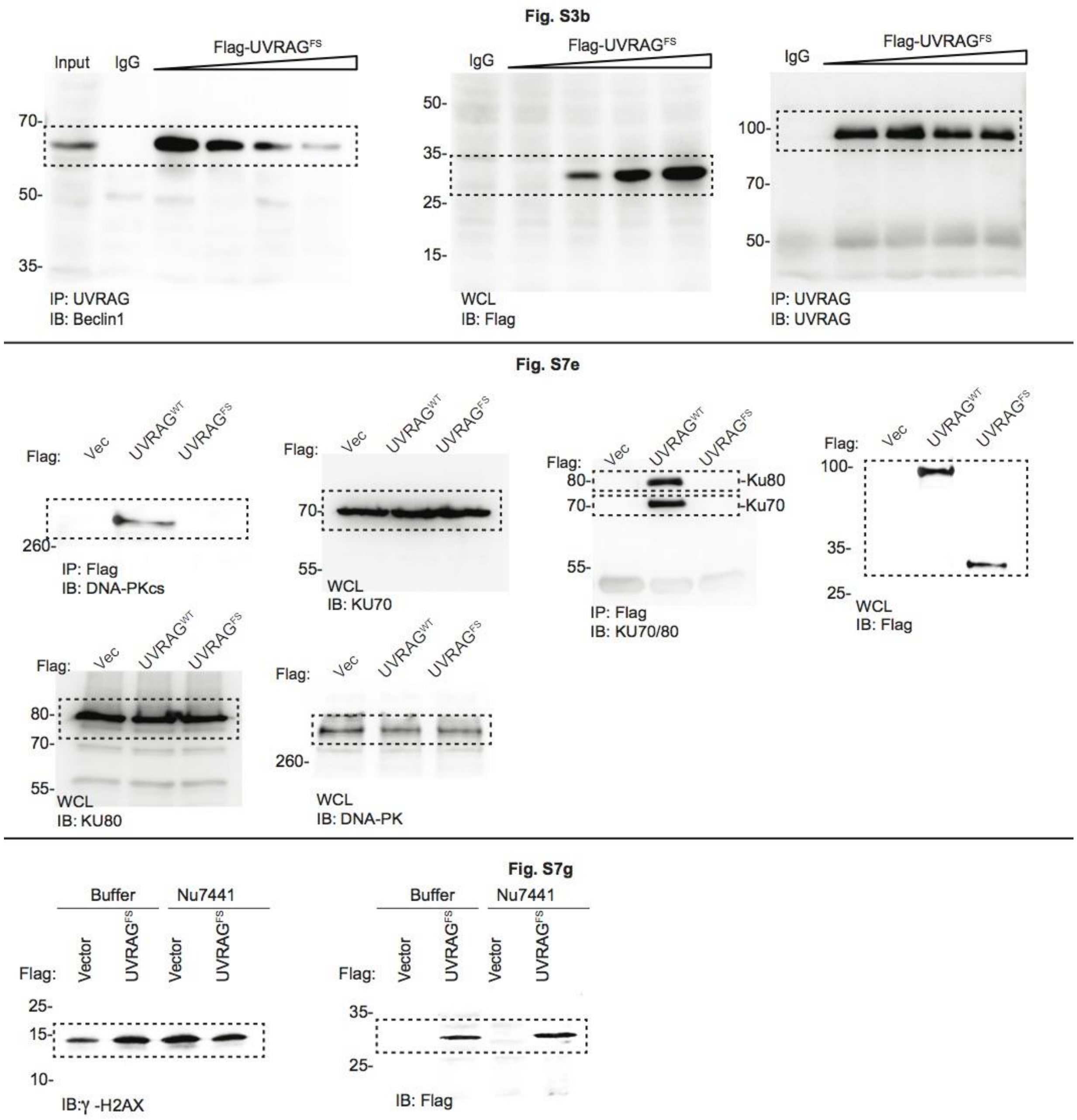

Supplementary Fig. 10

Supplementary Figure 10. The uncropped blots showing the experiments displayed in the main and supplementary figures

Supplementary Table 1. Clinicopathological features of MSS and MSI CRC patients.

Sample ID	Gender	Age	Microsatellite instability status	Tumor site	T stage	N stage	M stage	UVRAG Genotype
CYS001	M	71	MSI	Sigmoid colon	T3	N0	Mx	FS[a]
CYS002	F	73	MSI	Sigmoid colon	T3	N0	Mx	WT
CYS003	F	66	MSI	Right colon	T2	N1a	Mx	WT
CYS004	M	52	MSI	Rectum	T2	N0	Mx	WT
CYS005	F	41	MSS	Sigmoid colon	N/A	N1a	Mx	WT
CYS006	M	50	MSS	Rectosigmoid colon	T3	N0	Mx	WT
CYS007	M	54	MSS	Rectum	T3	N0	Mx	WT
CYS008	F	67	MSS	Right colon	T2	N0	Mx	WT

[a] an A deletion in the A_{10} repeat of the exon 8 of UVRAG.

Supplementary Reference:

1. Wang, K. *et al.* Whole-genome sequencing and comprehensive molecular profiling identify new driver mutations in gastric cancer. *Nat Genet* **46**, 573-82 (2014).
